# Supplementary material for: Metabolic dysregulation in vitamin E and carnitine shuttle energy mechanisms associate with human frailty
Source: Nat Commun. 2019 Nov 5;10:5027. doi: 10.1038/s41467-019-12716-2 (PMC6831565; doi:10.1038/s41467-019-12716-2)
Supplement: Supplementary file 1 — Supplementary Information [file 41467_2019_12716_MOESM1_ESM.pdf]

# SUPPLEMENTARY INFORMATION

## Metabolic dysregulation in Vitamin-E and carnitine shuttle energy mechanisms associate with human frailty

Rattray et al.

## Supplementary Methods

Concentrations of serum based HDL cholesterol and triglycerides were measured using a Roche Modular Analyzer. High sensitivity C-reactive protein concentration was determined using Roche Modular P. The measurement of all biomarkers have been described in a number of official ELSA technical reports[1,2].

Cholesterol esters in each serum sample were hydrolysed by cholesterol esterase (CHE). The free cholesterol produced was oxidised by cholesterol oxidase (CHO) to cholestene-3-one with the simultaneous production of hydrogen peroxide ($H_2O_2$). Subsequently oxidative coupling with 4-aminophenazone and phenol in the presence of peroxidase (POD) yielded Quinoneimine, a chromophore that was measured spectrophotometrically at 505 / 700 nm as an increase in absorbance. In the presence of $Mg^{2+}$, dextran sulfate selectively formed water-soluble complexes with LDL which are resistant to PEG-modified enzymes. The cholesterol concentration of HDL-cholesterol was determined enzymatically by cholesterol esterase and cholesterol oxidase coupled with PEG to associated amino groups (approx. 40%). Cholesterol esters were broken down quantitatively into free cholesterol and fatty acids by cholesterol esterase. In the presence of oxygen, cholesterol was oxidized by cholesterol oxidase to Δ4-cholestenone and hydrogen peroxide. In the presence of this peroxidase, hydrogen peroxide generated reacted with 4-amino-antipyrine and HSDA to form a purple-blue dye. The color intensity of this dye was measured bichromatically at 600 / 700 nm and assumed directly proportional to the cholesterol concentration. Similarly, triglycerides were hydrolysed by a combination of microbial lipases to yield glycerol and fatty acids. Glycerol was phosphorylated by adenosine triphosphate(ATP) in the presence of glycerol kinase (GK) to produce glycerol-3-phosphate. Glycerol-3-phosphate was oxidised by molecular oxygen in the presence of glycerol phosphate oxidase (GPO) to produce hydrogen peroxide ($H_2O_2$) and dihydroxyacetone phosphate. The formed $H_2O_2$ reacted with 4-aminophenazone and 4-chlorophenol in the presence of peroxidise to produce a chromophore. The increase in absorbance at 505 / 700 nm was measured proportional to the triglyceride content of the sample.

Measurement of Insulin like Growth Factor 1 (IGF-1) was made by an IDS iSYS immunoassay analyser assay. Samples were incubated under acidic conditions to dissociate IGF-I from the binding proteins. A portion of this, along with neutralisation buffer, a biotinylated anti-IGF-I monoclonal antibody and an acridinium labelled anti IGF-I monoclonal antibody were incubated together. Streptavidin labelled magnetic particles were then added and following a further incubation step, the magnetic particles were captured. After washing, trigger reagents were added and the light emitted was directly proportional to the [IGF-I] in the original sample. The assay represents a new generation of assay which was calibrated to the new WHO international standard for insulin-like Growth Factor-I, NIBSC code: 02/254. The assay also conforms to the 2011 consensus statement on GH and IGF-I assays.

Measurement of HbA1c were made using a Glycohemoglobin Analyser TOSOH HLC-723G8. Ion exchange high performance liquid chromatography (HPLC) was used for the automatic separation of haemoglobin A1c (HbA1c). Whole blood was sampled directly from the primary tube, diluted in haemolysing buffer and pumped via a pre-filter to the ion-exchange column. A series of three G8 Variant elution buffers of differing ionic strengths provide a stepped gradient with which the haemoglobin fractions were eluted from the column. There was no pre-treatment

of the sample to remove the labile A1c as this elutes as a separate peak. Peaks were detected by dual wavelength measurement and quantitated using calibrators aligned to IFCC values.

Measurement of Haemoglobin was performed on a Sysmex XE-2100 Analyser. Whole blood was aspirated to the rotor valve. 3.0 µL of whole blood was then diluted to a ratio of 1:333 with 0.9970 mL of the isotonic Cellpack reagent. This was then sent to the flowcell. At the same time 0.5 mL of Sulfolyser was added to haemolyse the red blood cells to make a 1:500 diluted sample, and the haemoglobin was converted to SLS_haemoglobin. The concentration of haemoglobin was subsequently measured as light absorbance (at 555 nm) and was calculated by comparison with the absorbance of the diluent measured before the sample was added.

Measurement of Fibrinogen was performed the on an ACL TOP CTS analyser. A 1 in 10 dilution of plasma in Factor diluent was made by aspirating 17 µL of plasma and mixing it with 153 µL of Factor Diluent. 100 µL of the diluted plasma was then dispensed into a reaction cuvette and incubated for 60 s at 37°C. 50 µL of Fibrinogen-C XL reagent was subsequently added and the reaction monitored at 405 nm for 120 s. The change in light transmission caused by the conversion of soluble Fibrinogen in plasma to cross-linked insoluble Fibrin was monitored by the analyser and the clotting time threshold was determined to be 37% of the total change. As the clotting time is directly related to the concentration of Fibrinogen in the plasma the time was converted to concentration in g/L by automatic use of a calibration curve.

**Supplementary Figures**

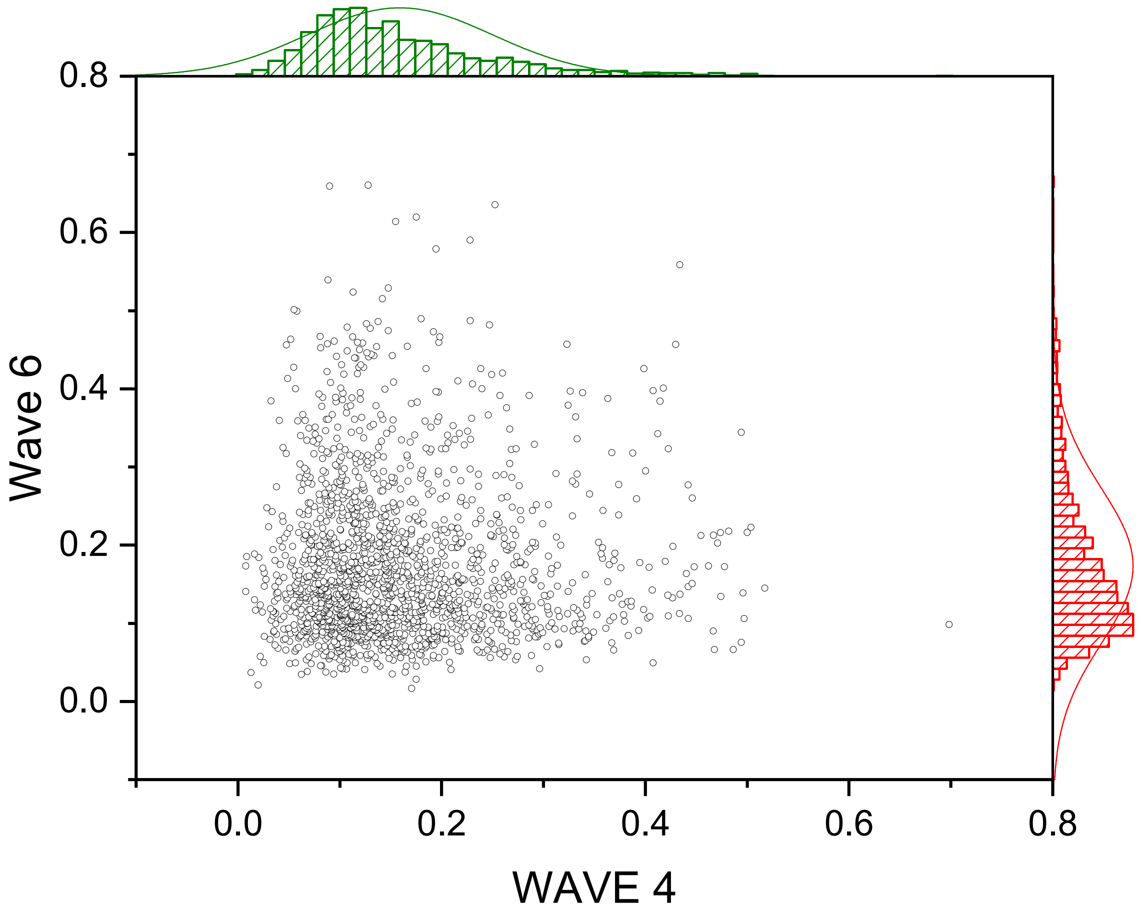


**Supplementary Figure 1|** Frailty indices produced from 1843 subjects within Wave 4 and 1753 subjects within Wave 6 of the English Longitudinal Study of Ageing. The observed unimodal right skewed distributions are comparable to those produced in other population scale assessments of frailty. Black circles represent the frailty index value of each subject. Green distribution = Wave 4. Red Distribution = Wave 6. Source data are provided as a Source Data file.

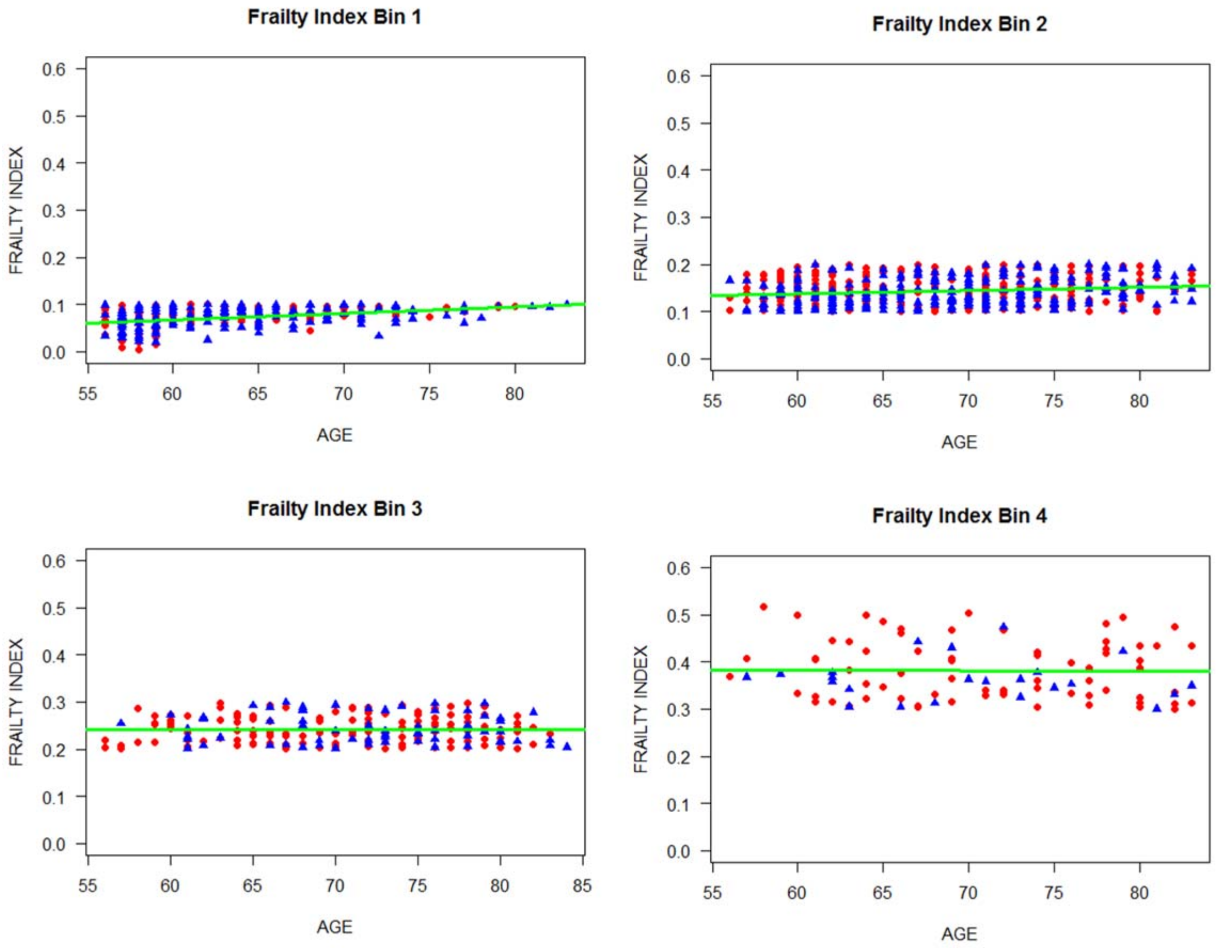


| Index | Regression Equation | Pearson's ***R*** Correlation | $R^2$ |
|---|---|---|---|
| **F1** | F1 = 0.00141 (AGE) – 0.017 | 0.4103758 | 0.1684083 |
| **F2** | F2 = 0.1027842 (AGE) + 0.0005897 | 0.1386819 | 0.01923266 |
| **F3** | F3 = $2.366e^{-1}$ (AGE) + $7.759e^{-5}$ | 0.01908764 | 0.0003643379 |
| **F4** | F4 = 0.4242092 (AGE) -0.0006516 | -0.07644561 | 0.005843931 |
| **FULL** | F_Total = -0.119336 (AGE) + 0.004092 | 0.335749 | 0.1127274 |

**Supplementary Figure 2|** Alongside the full frailty index linear regression model with 1191 subjects (**Fig 1a**), analyses were also carried out on data from each frail level (blue triangles = male subjects, red dots = female subjects). Regression summary table indicates the Pearson's R Correlation between frailty and age lowers as FI score increases. Source data are provided as a Source Data file.

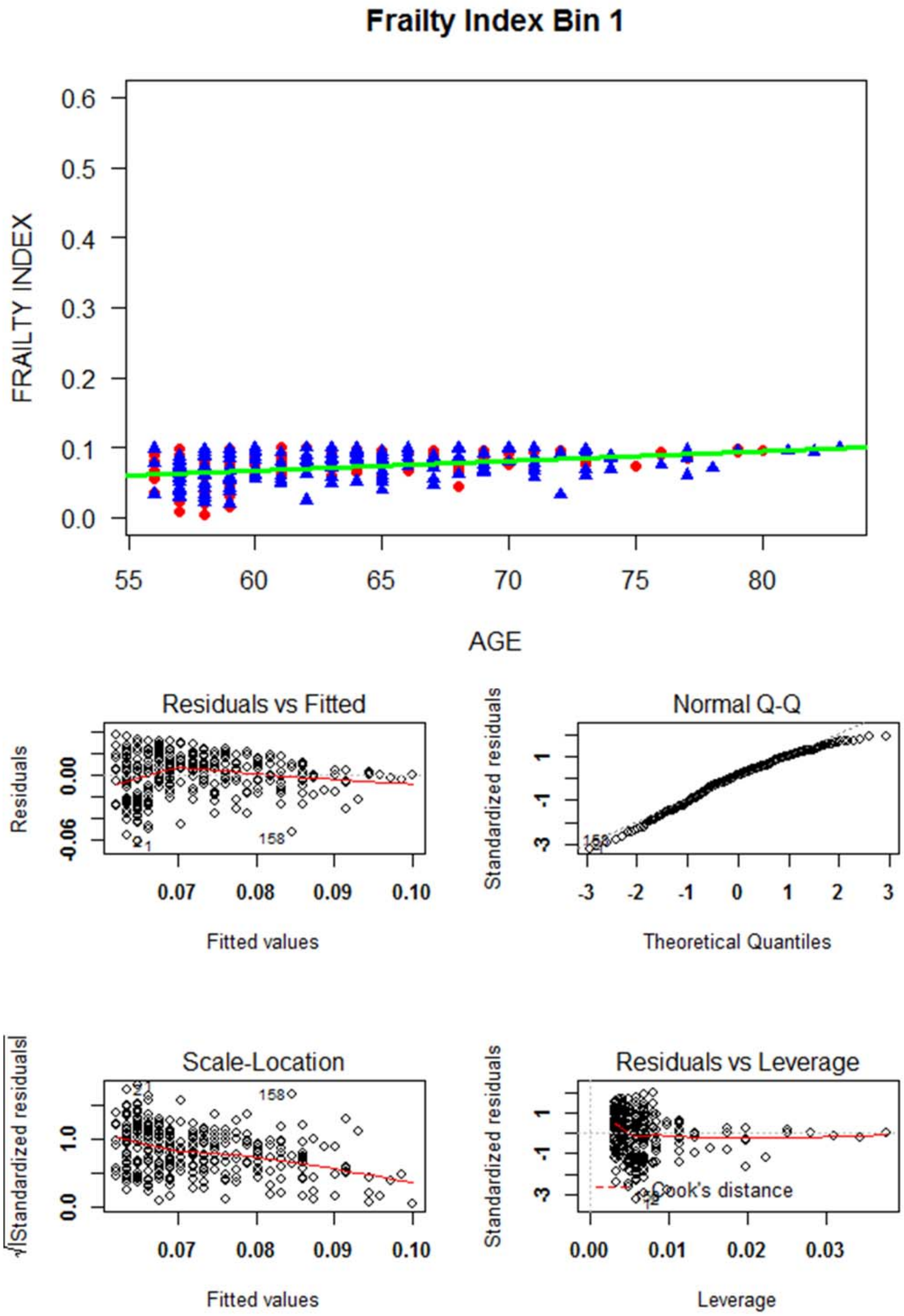


**Supplementary Figure 3|** Regression plots of Frailty Index data bin 1 (blue triangles = male subjects, red dots = female subjects). Source data are provided as a Source Data file.

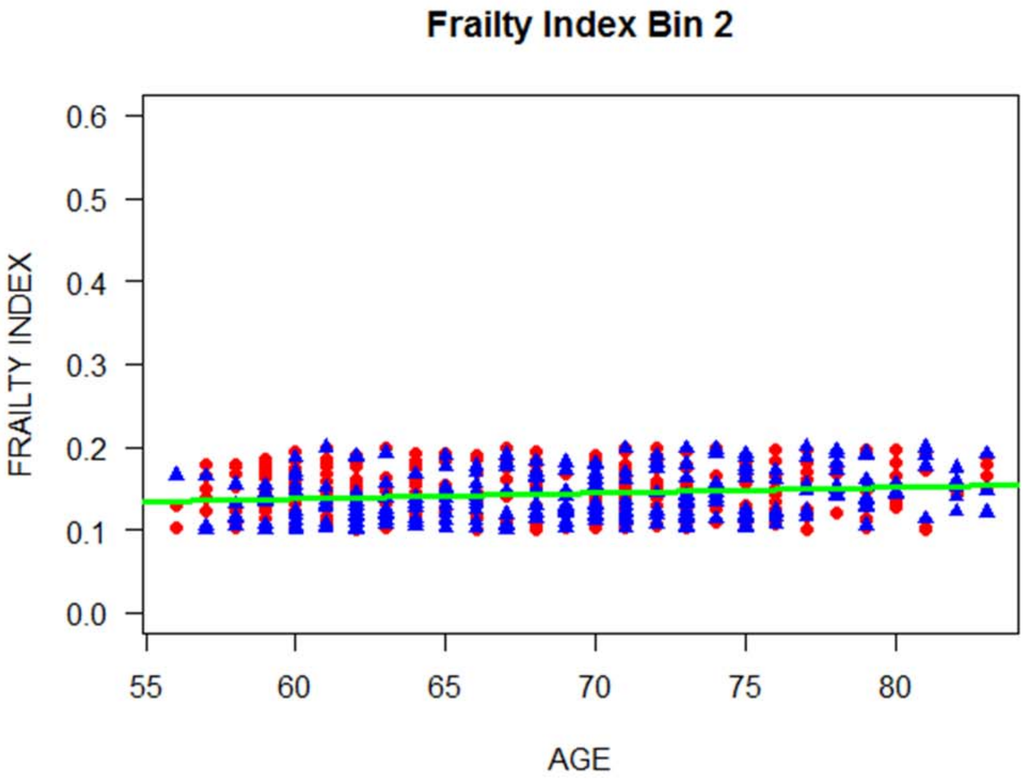


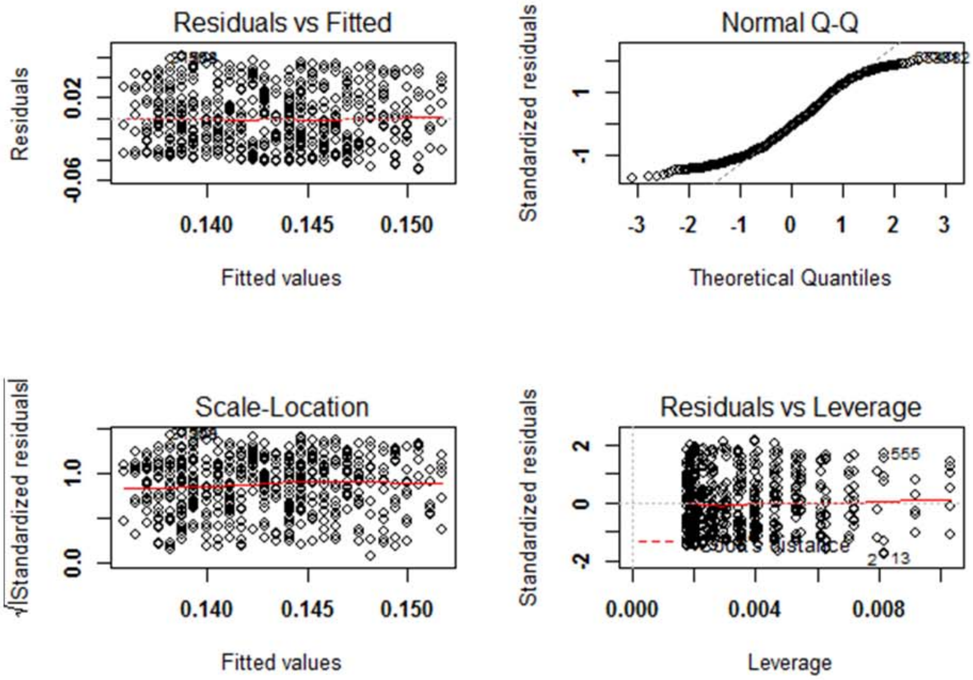


**Supplementary Figure 4|** Regression plots of Frailty Index data bin 2 (blue triangles = male subjects, red dots = female subjects). Source data are provided as a Source Data file.

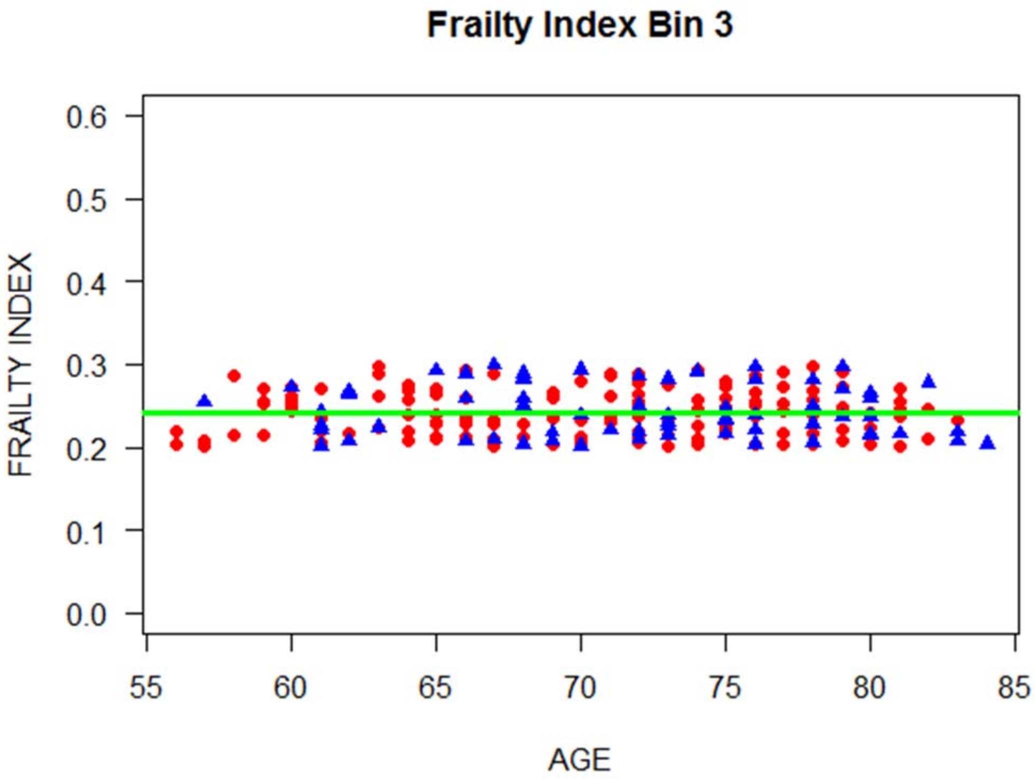


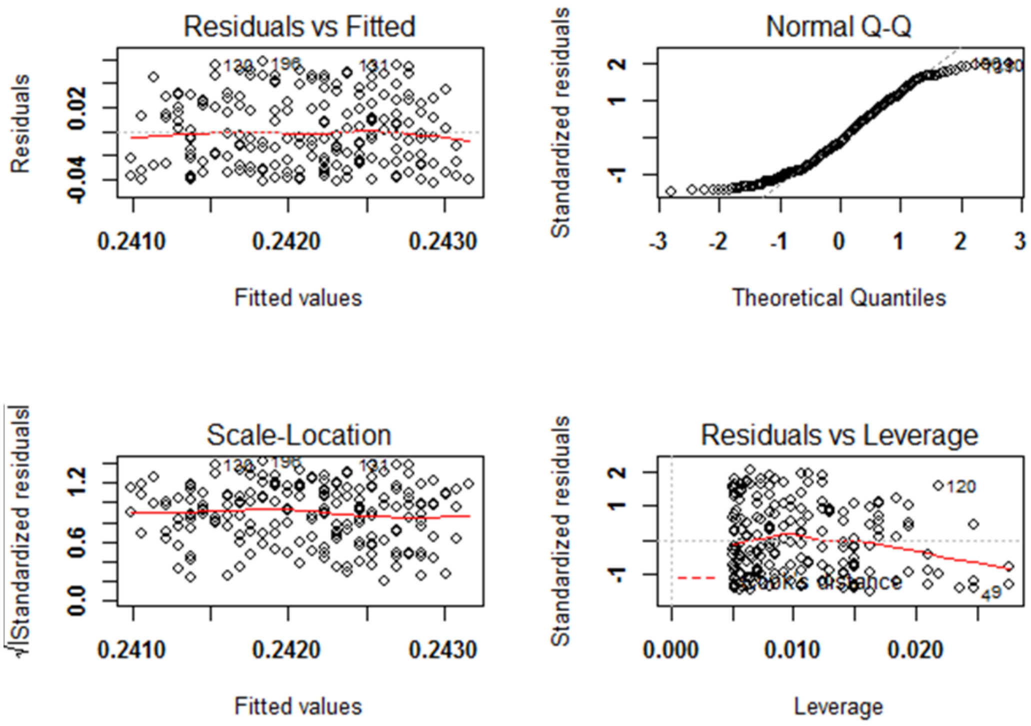


**Supplementary Figure 5|** Regression plots of Frailty Index data bin 3 (blue triangles = male subjects, red dots = female subjects). Source data are provided as a Source Data file.

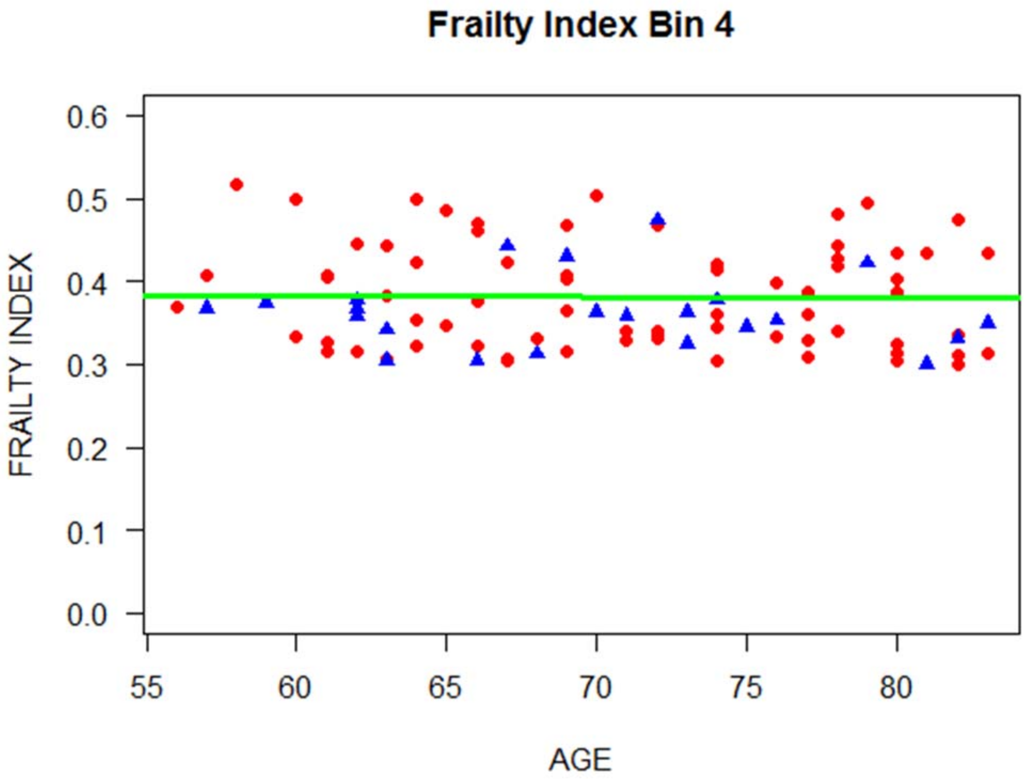


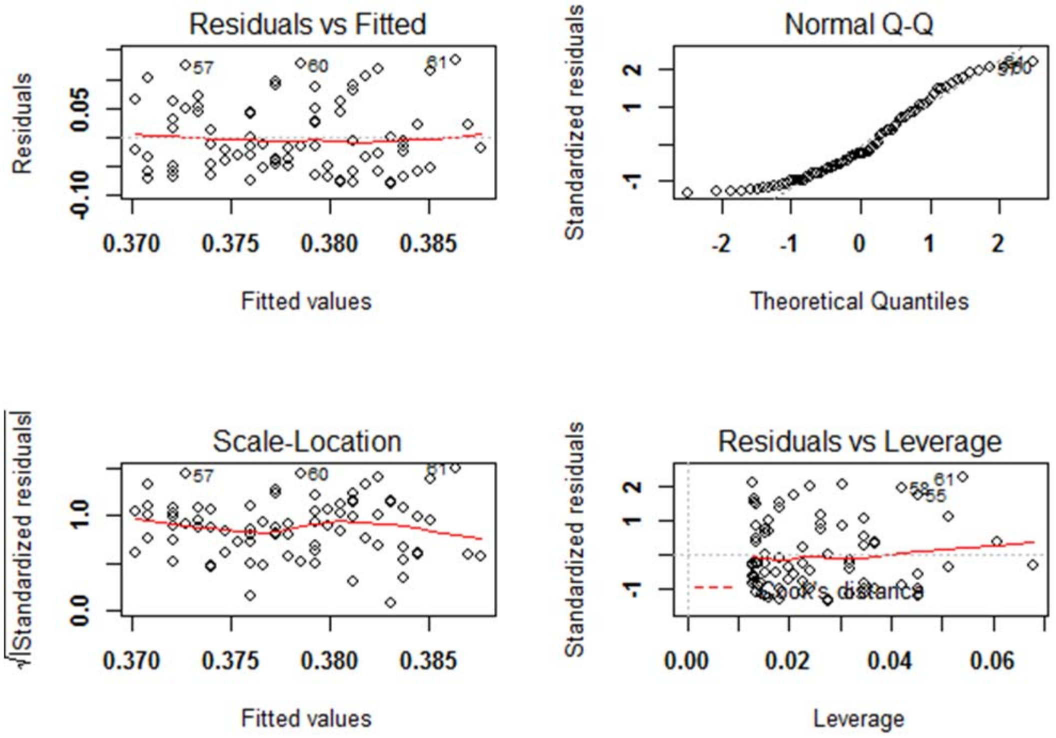


**Supplementary Figure 6|** Regression plots of Frailty Index data bin 4 (blue triangles = male subjects, red dots = female subjects). Source data are provided as a Source Data file.

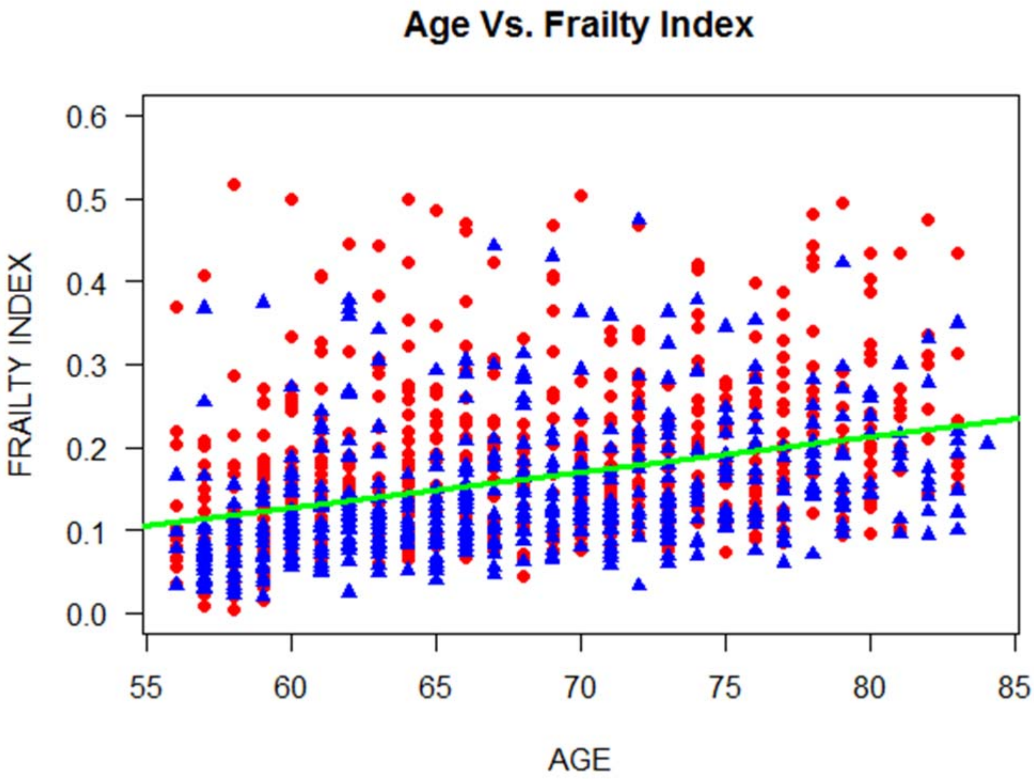


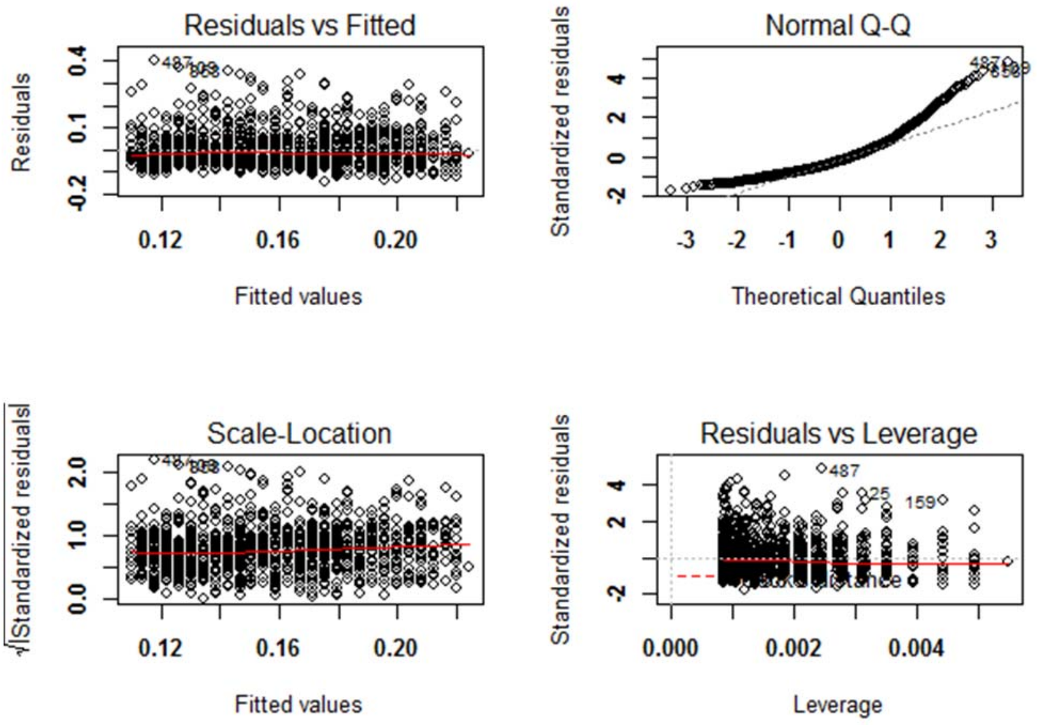


**Supplementary Figure 7|** Regression plots of All Frailty Index data bins combined (blue triangles = male subjects, red dots = female subjects). Source data are provided as a Source Data file.

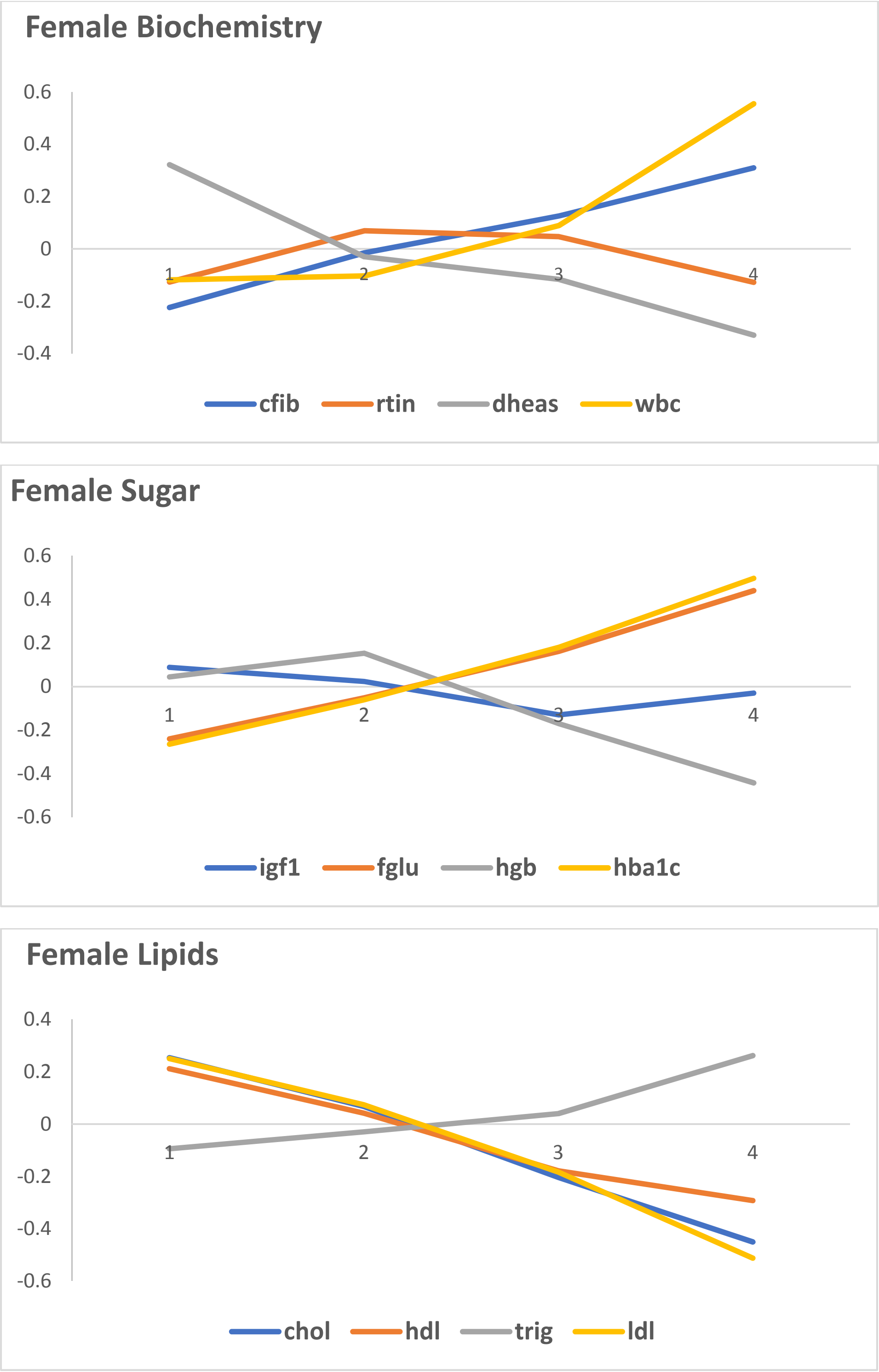


**Supplementary Figure 8|** Female sex stratification of biochemistry, carbohydrate and lipid z-score data vs. frail index. Source data are provided as a Source Data file.

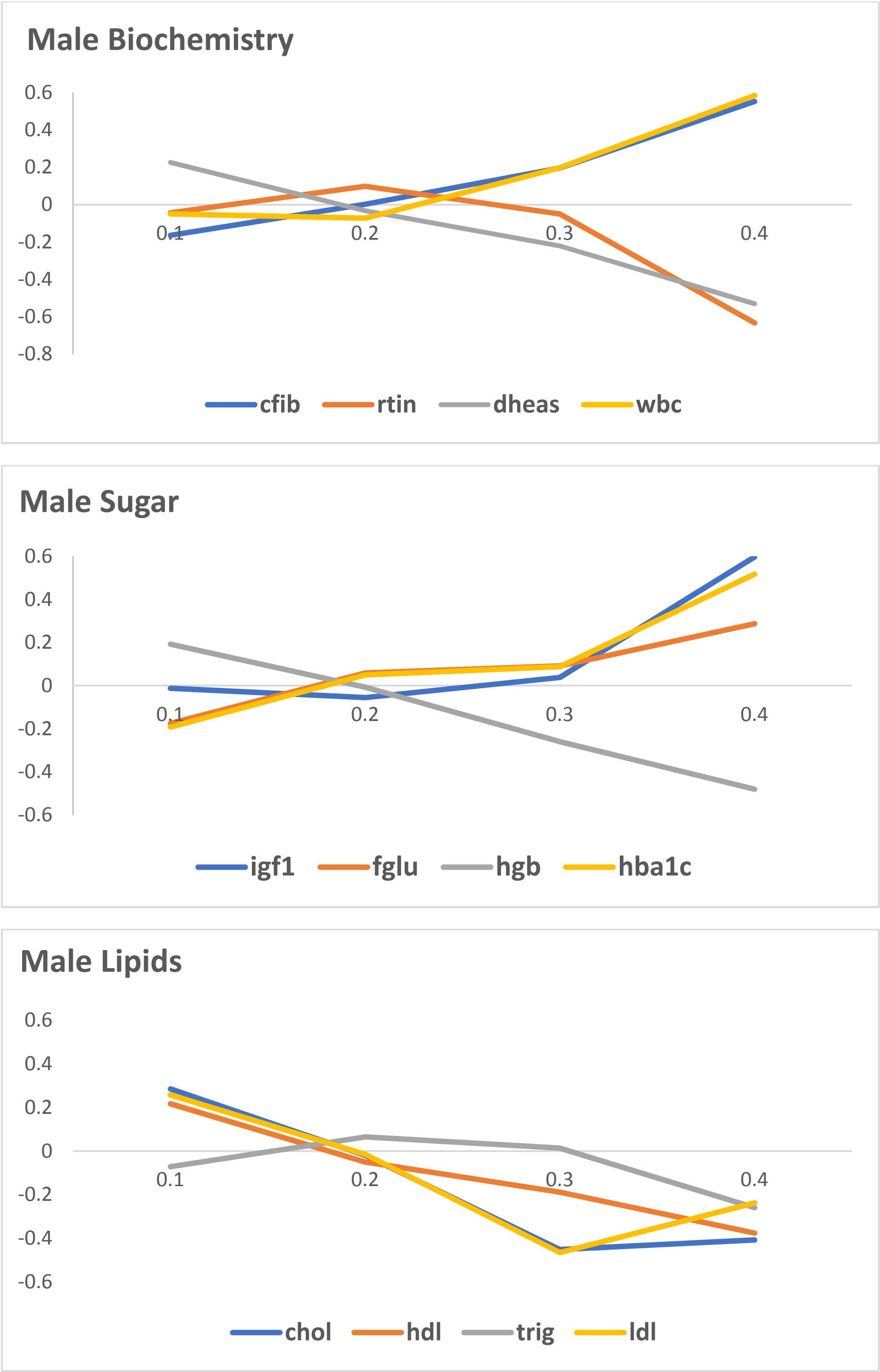


**Supplementary Figure 9|** Male Sex stratification of biochemistry, carbohydrate and lipid z-score data vs. frail index bin. Source data are provided as a Source Data file.

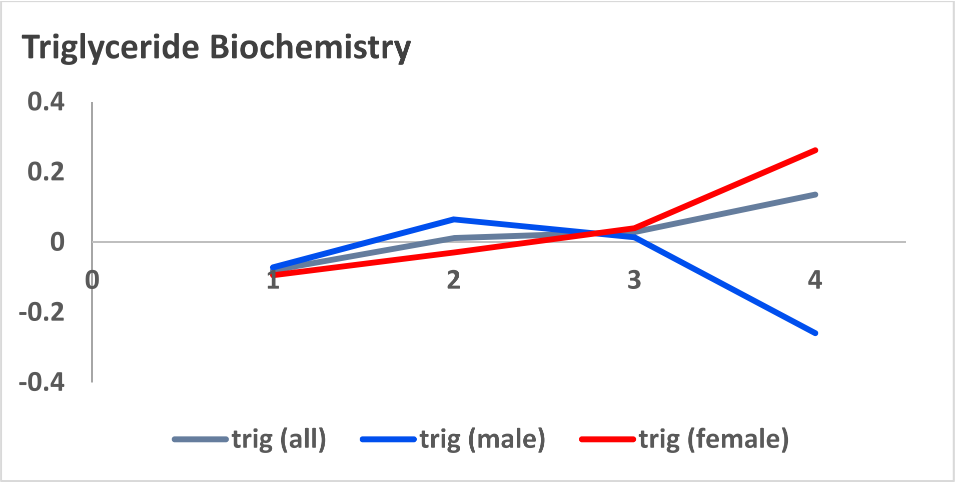


**Supplementary Figure 10|** Sex stratification of triglyceride z-score data vs. frail index bin. It is noted that there is divergence of triglyceride levels upon comparison at higher frailty index values. Source data are provided as a Source Data file.

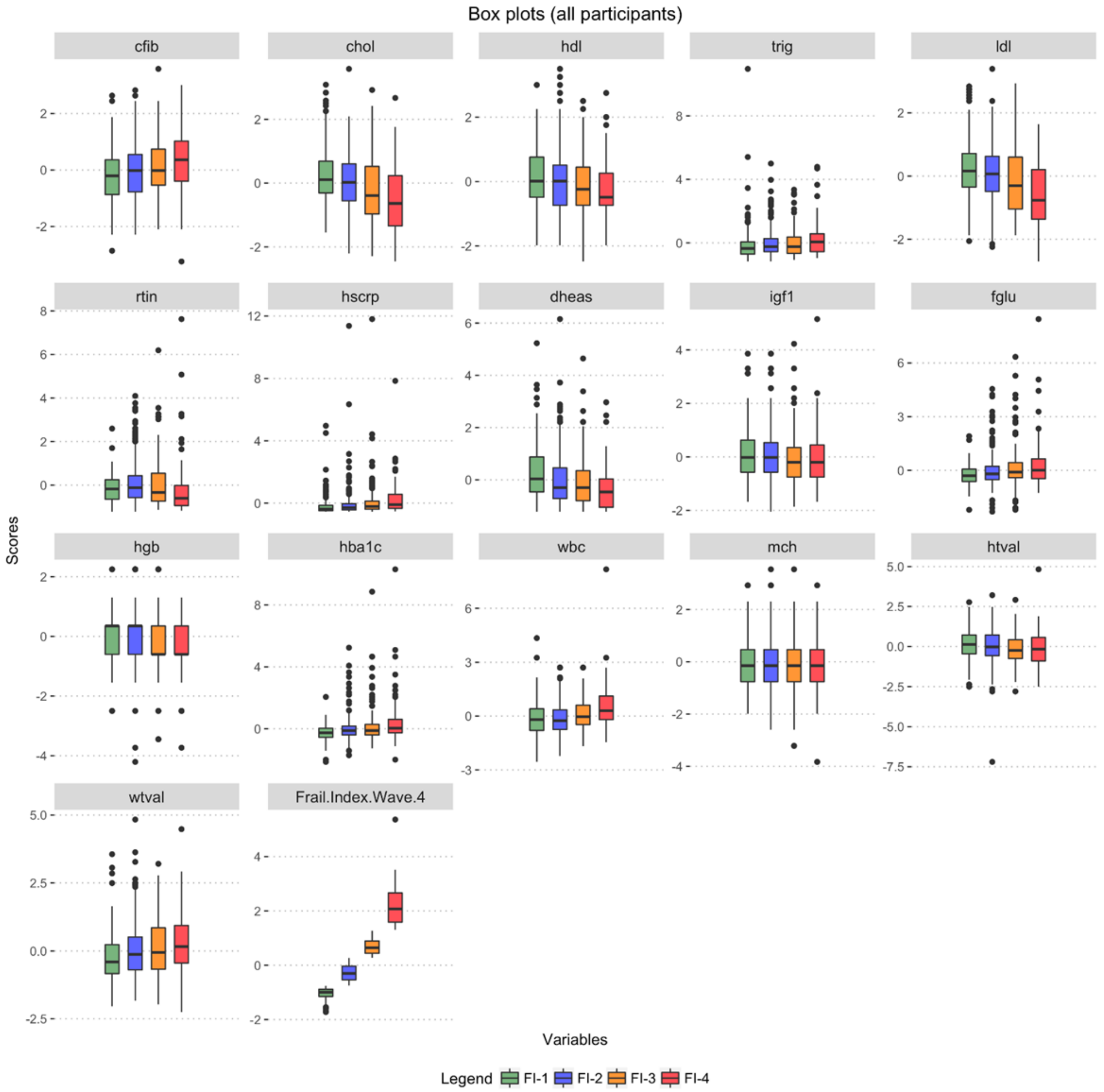


**Supplementary Appendix 11|** Boxplots of all Wave 4 biochemistry measurements measured against frailty index bin. Each boxplot displays median value (center line), upper and lower quartiles (box limits) and points out of interquartile range are outliers. Green Boxplot = Frailty Index 0 – 0.1, Blue Boxplot = Frailty Index 0.1 – 0.2, Orange Boxplot = Frailty Index 0.2 – 0.3, Red Boxplot = Frailty Index 0.3 and above. Source data are provided as a Source Data file.

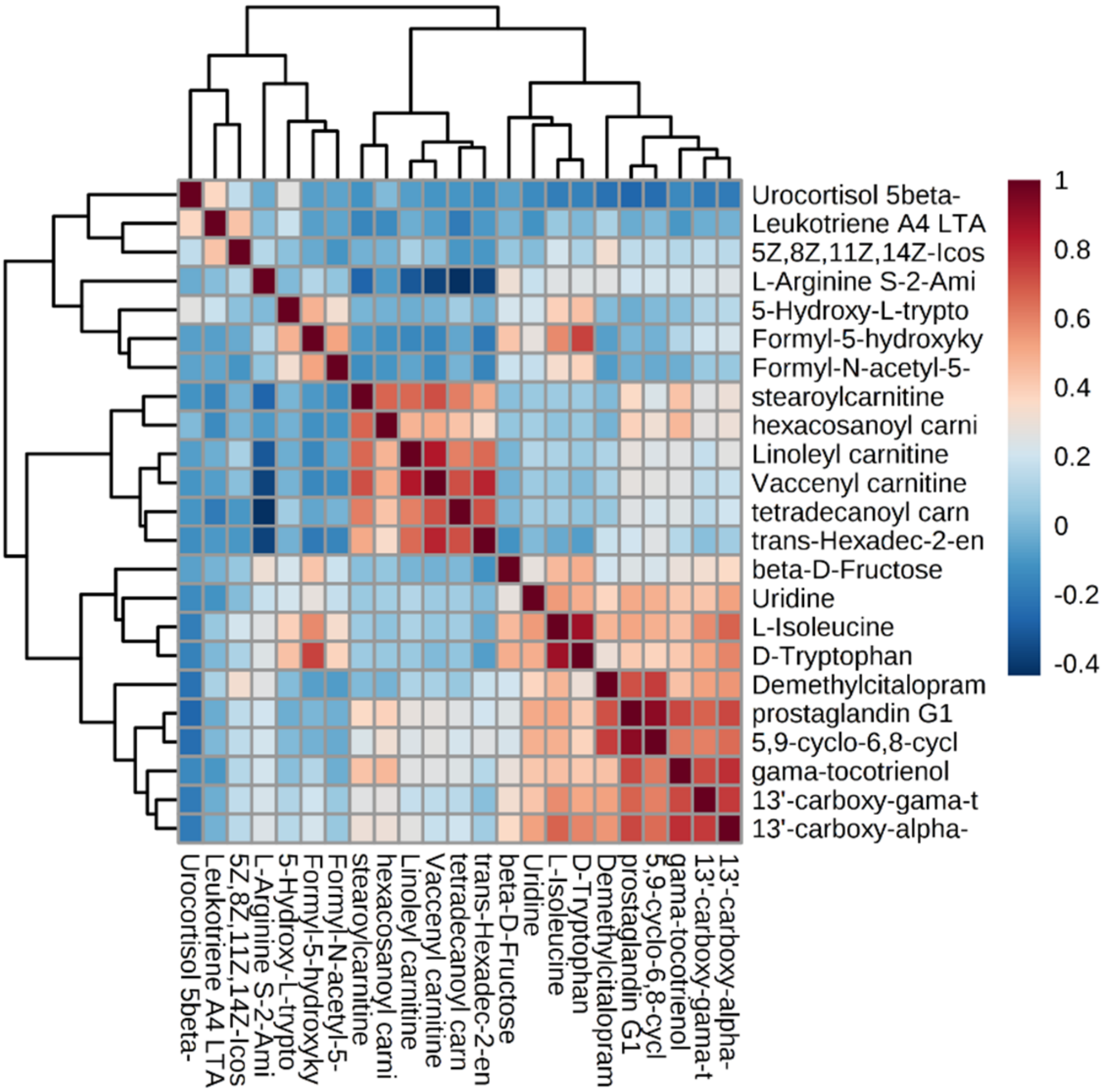


**Supplementary Figure 12|** Heat map of significant LCMS metabolites indicating the correlation between metabolites of similar classes linked to the up regulated pathways (Red colors indicate a higher correlation). Source data are provided as a Source Data file

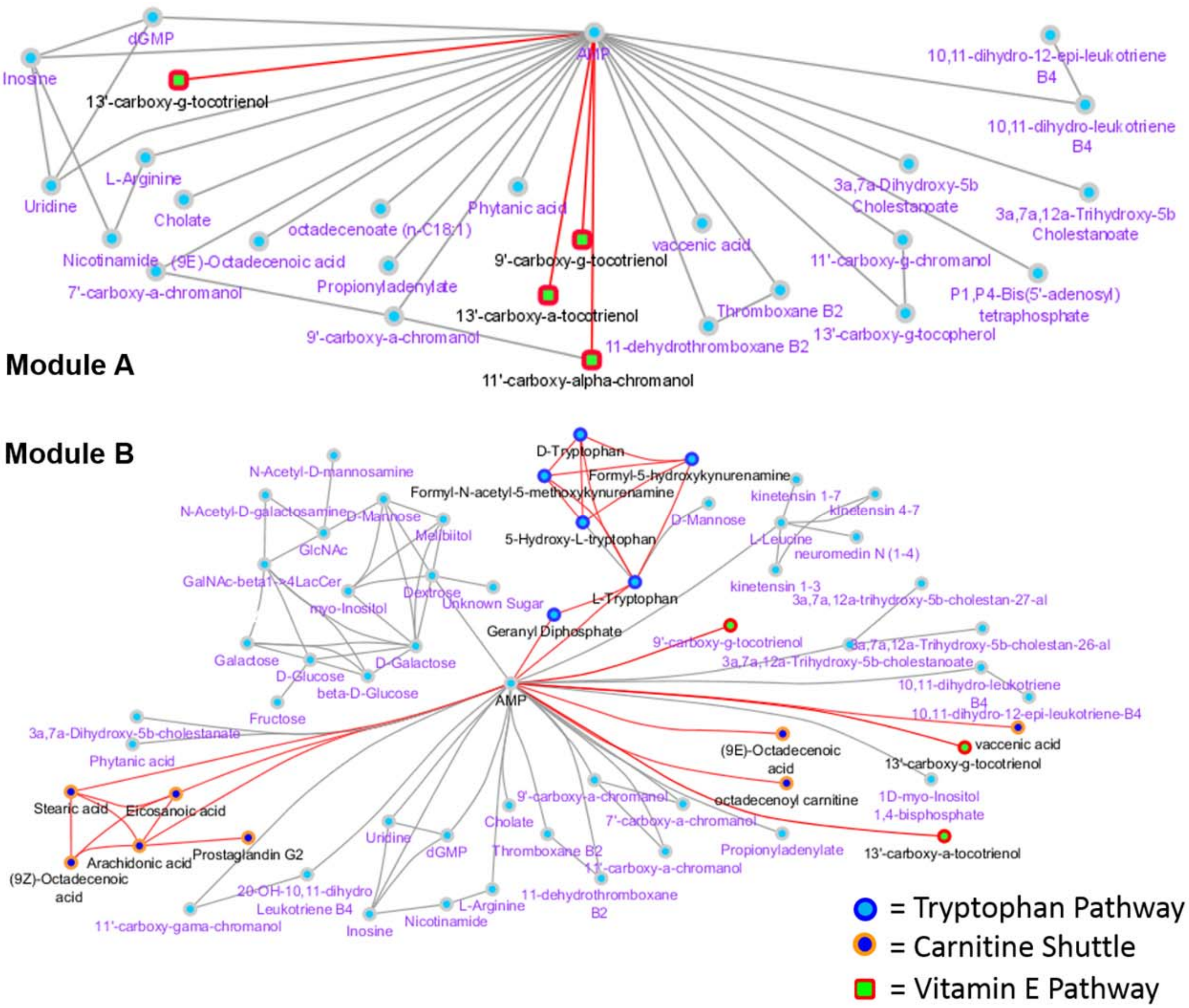


**Supplementary Figure 13|** Contributory metabolite modules identified in mummichog analysis highlighting three main pathways based on LCMS metabolite data. Module A highlights connectivity within the vitamin E pathway. Module B highlights connectivity with the tryptophan and carnitine shuttle pathways. Source data are provided as a Source Data file.

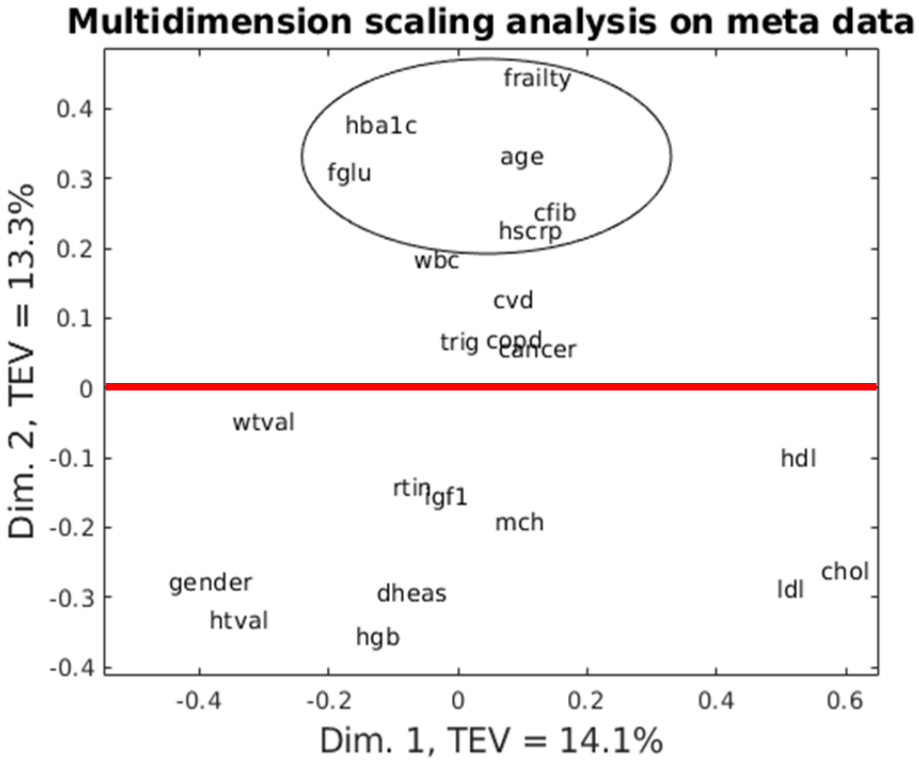


**Supplementary Figure 14|** Multidimensional Scaling Analysis used to measure the level of correlated (+ve value), or anti-correlated (-ve value) confounding factors related to frailty score (using 1 – Spearman Rho as the distance metric). Source data are provided as a Source Data file.

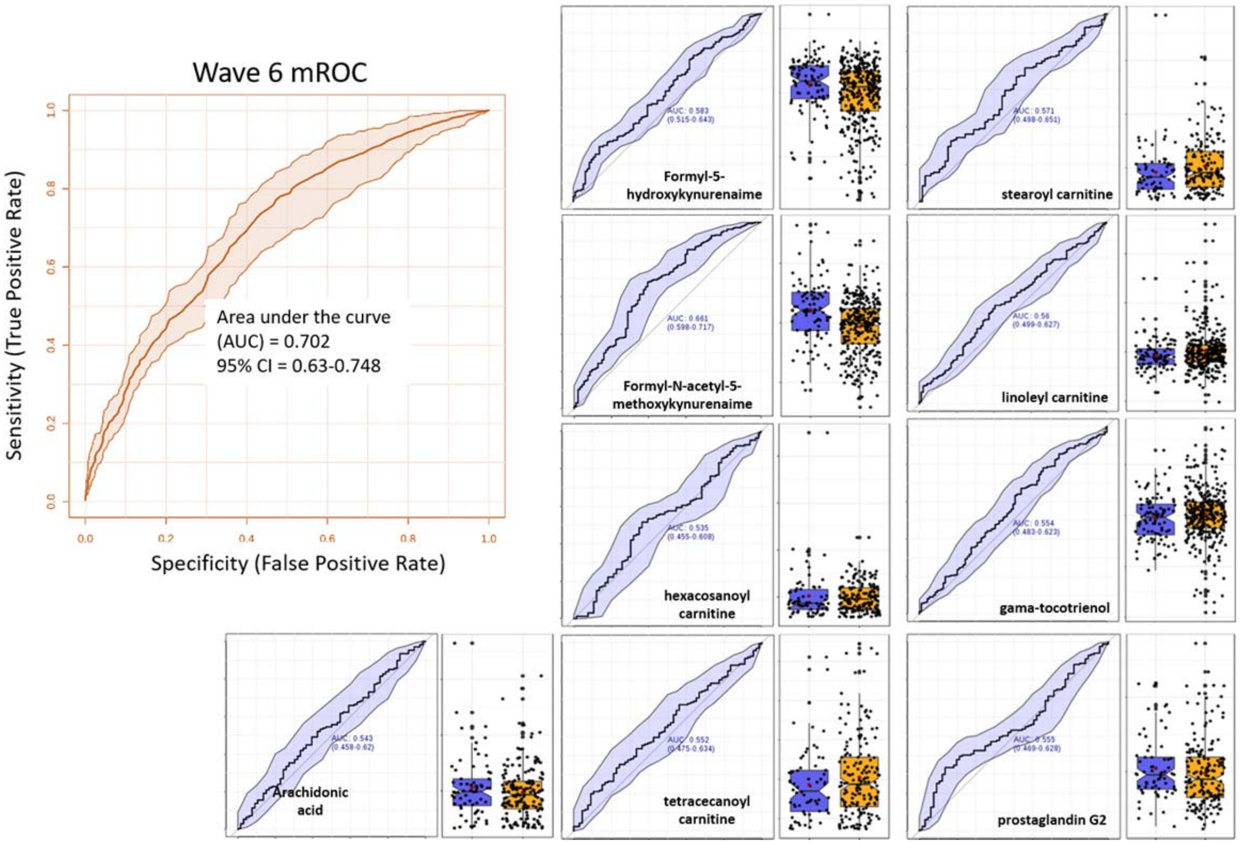


**Supplementary Figure 15|** Significance and diagnostic ability within the metabolic model of frailty. mROC curve from Waves 6 generated by combining 9 of the same significant metabolites detected in Wave 4 to generate a diagnostic model of frailty status. The shaded area indicate 95% confidence intervals calculated by Monte Carlo cross validation using balanced subsampling and 1000 iterations of bootstrapped cross-validation. Univariate ROC curves and non-frail (orange) to frail (blue) boxplots of each metabolite were used to generate the multivariate ROC analysis. Source data are provided as a Source Data file.

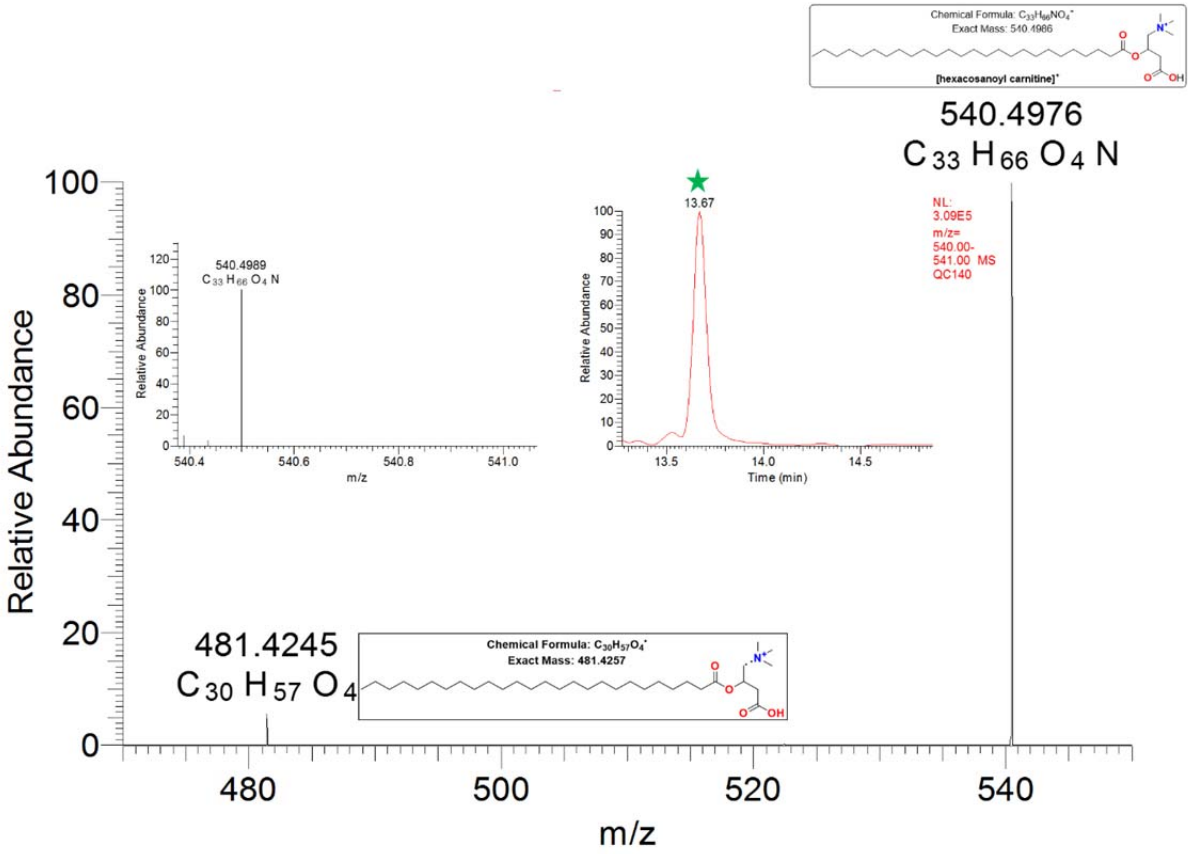


**Supplementary Figure 16|** Accurate mass and MSMS data on hexacosonyl-carnitine. Source data are provided within the data. Raw data used to generate spectra within this image are available within the associated MetaboLights Study upload (MTBLS598).

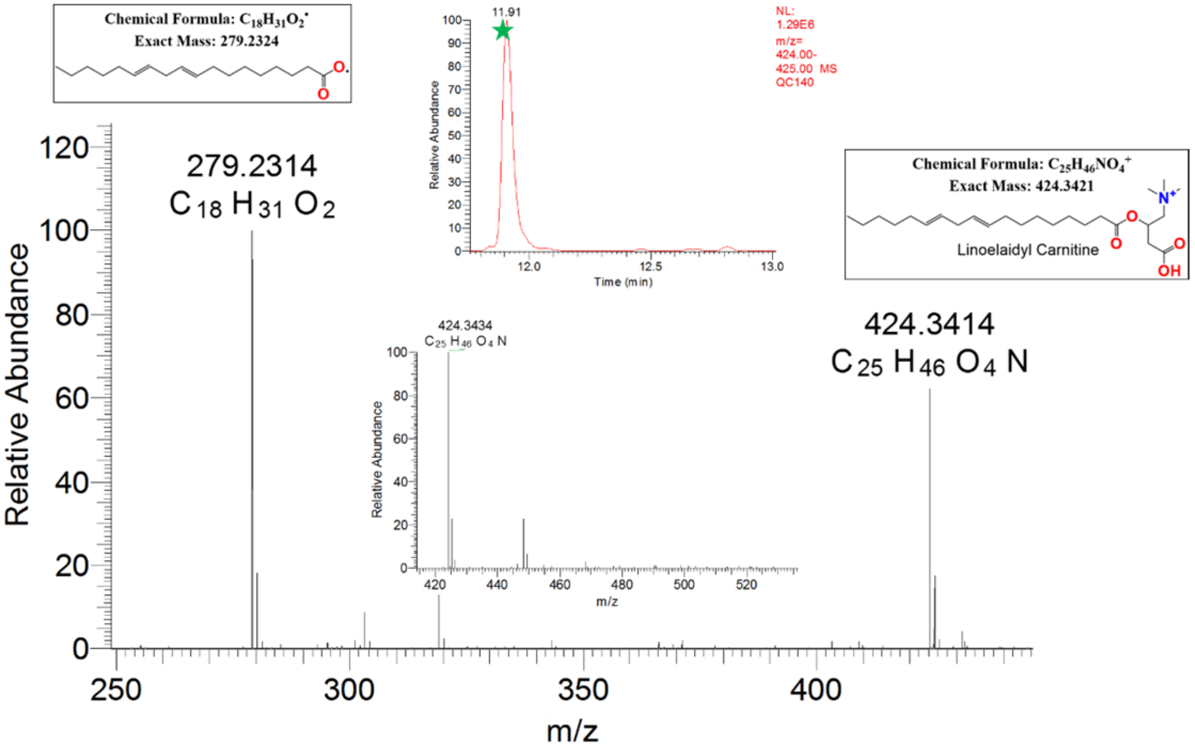


**Supplementary Figure 17|** Accurate mass and MSMS data on linoleyl carnitine. Raw data used to generate spectra within this image are available within the associated MetaboLights Study upload (MTBLS598).

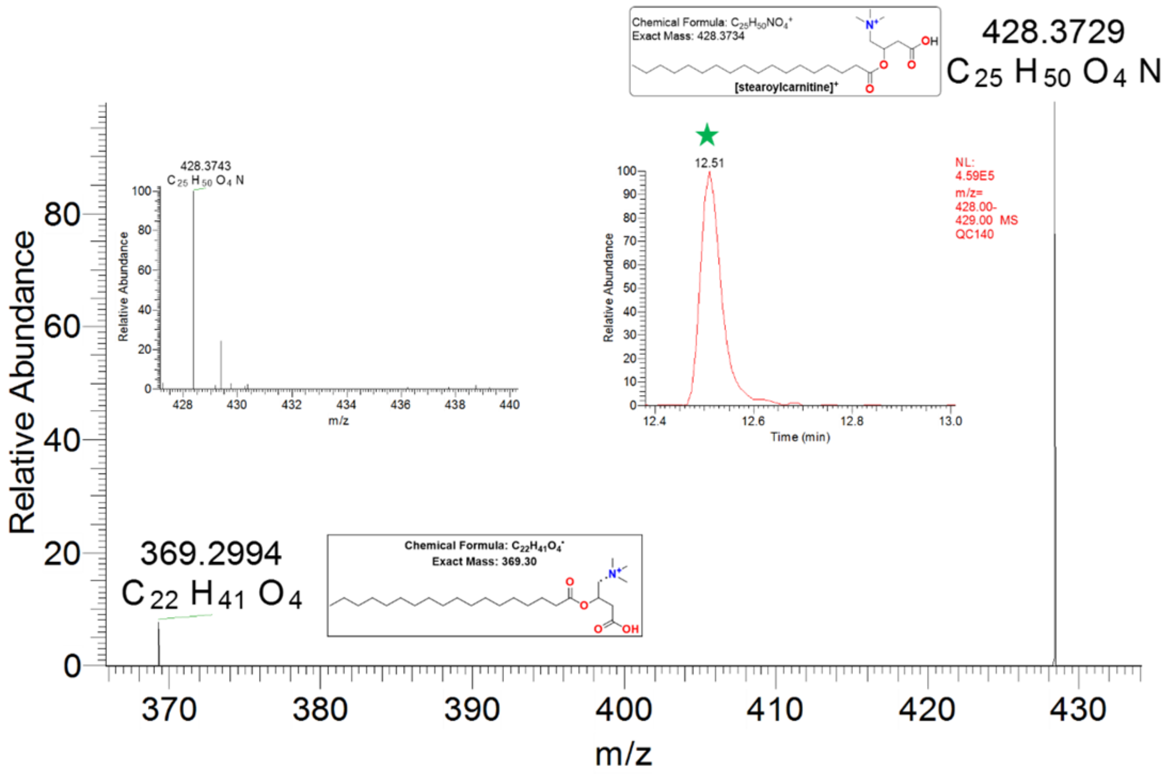


**Supplementary Figure 18|** Accurate mass and MSMS data on steroyl carnitine. Raw data used to generate spectra within this image are available within the associated MetaboLights Study upload (MTBLS598).

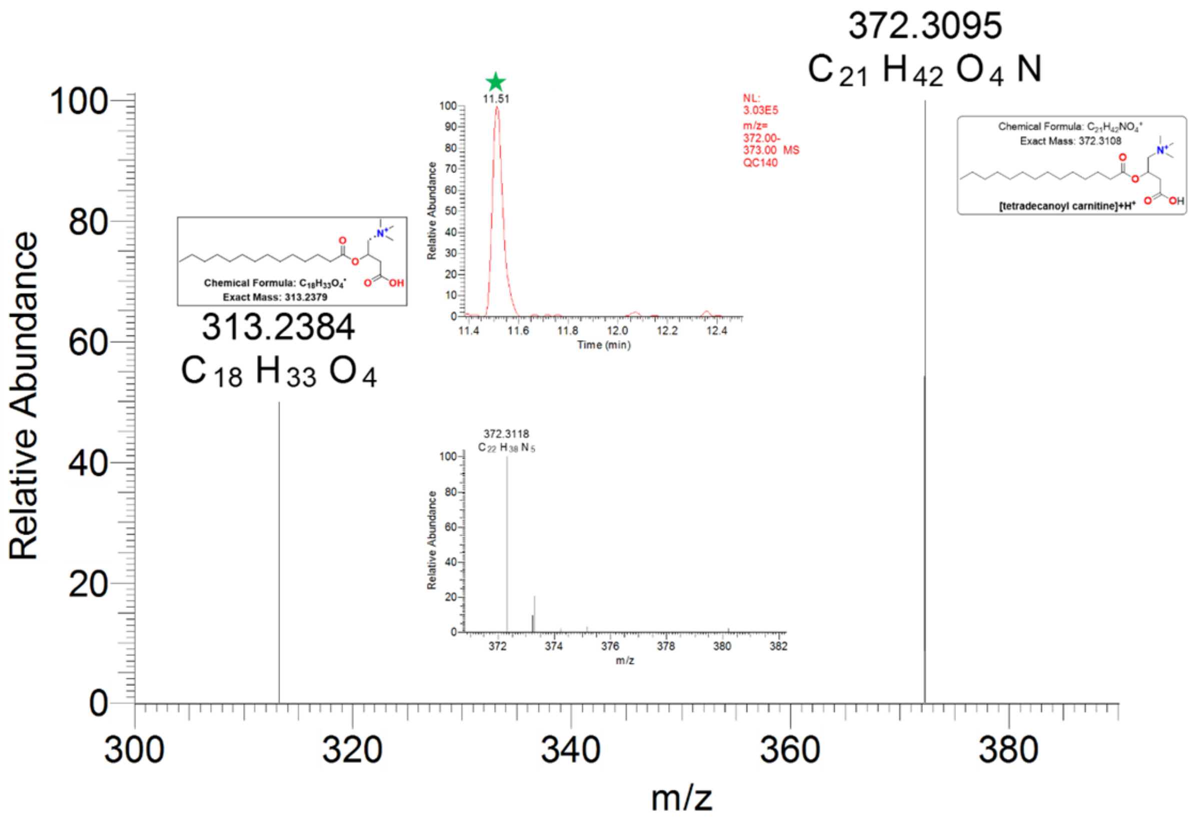


**Supplementary Figure 19|** Accurate mass and MSMS data on tetradeconoyl carnitine. Raw data used to generate spectra within this image are available within the associated MetaboLights Study upload (MTBLS598).

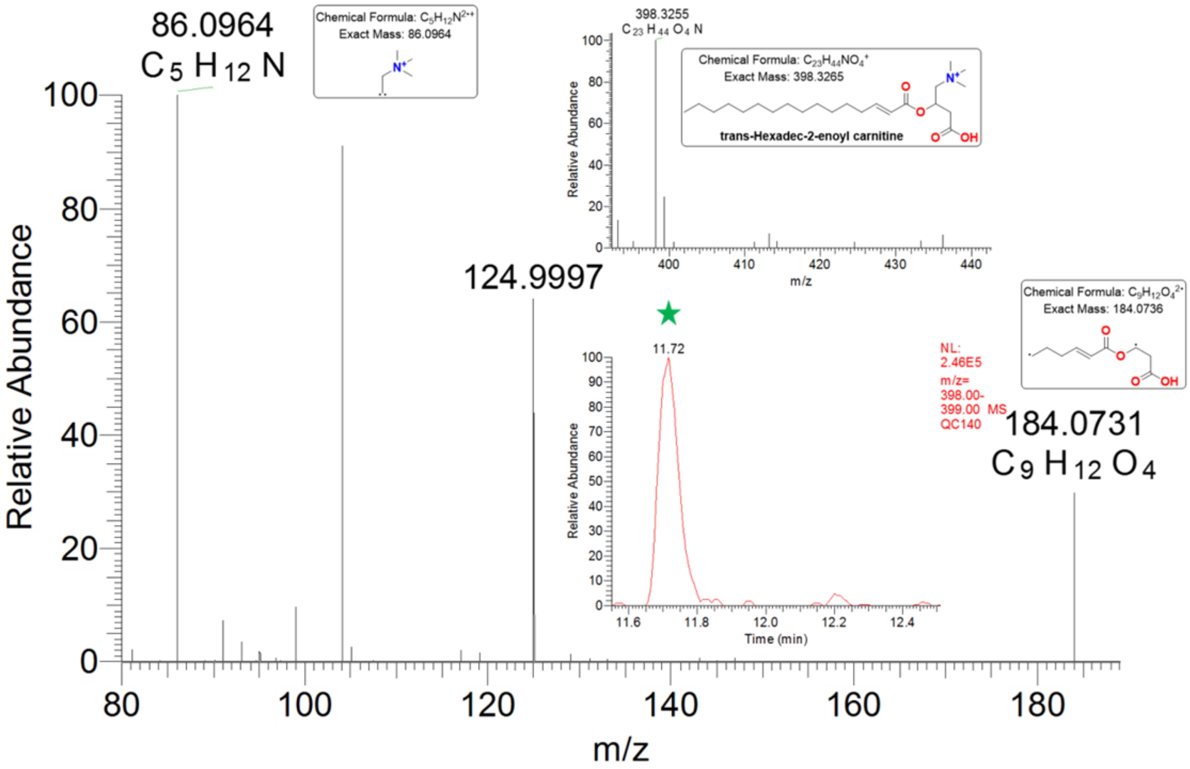


**Supplementary Figure 20|** Accurate mass and MSMS data on trans-hexadec-2-enoyl carnitine. Raw data used to generate spectra within this image are available within the associated MetaboLights Study upload (MTBLS598).

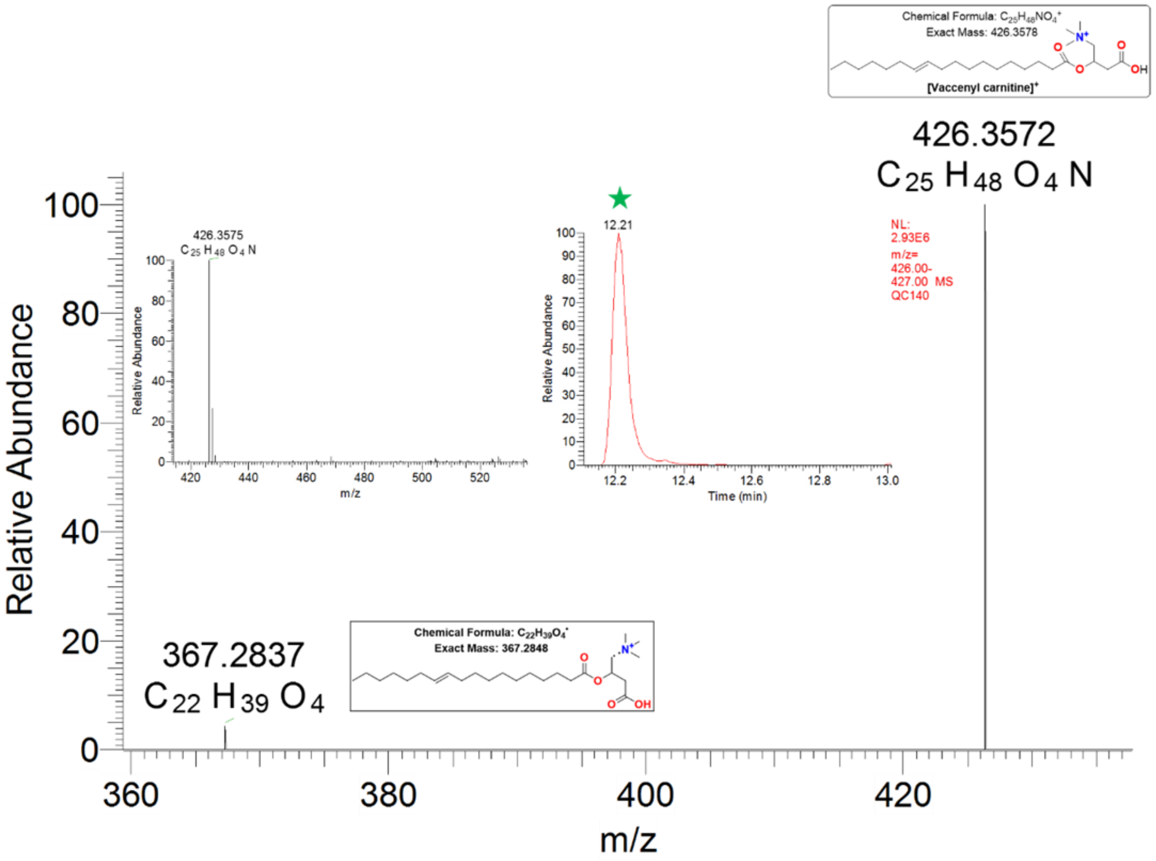


**Supplementary Figure 21|** Accurate mass and MSMS data on vaccenyl carnitine. Raw data used to generate spectra within this image are available within the associated MetaboLights Study upload (MTBLS598).

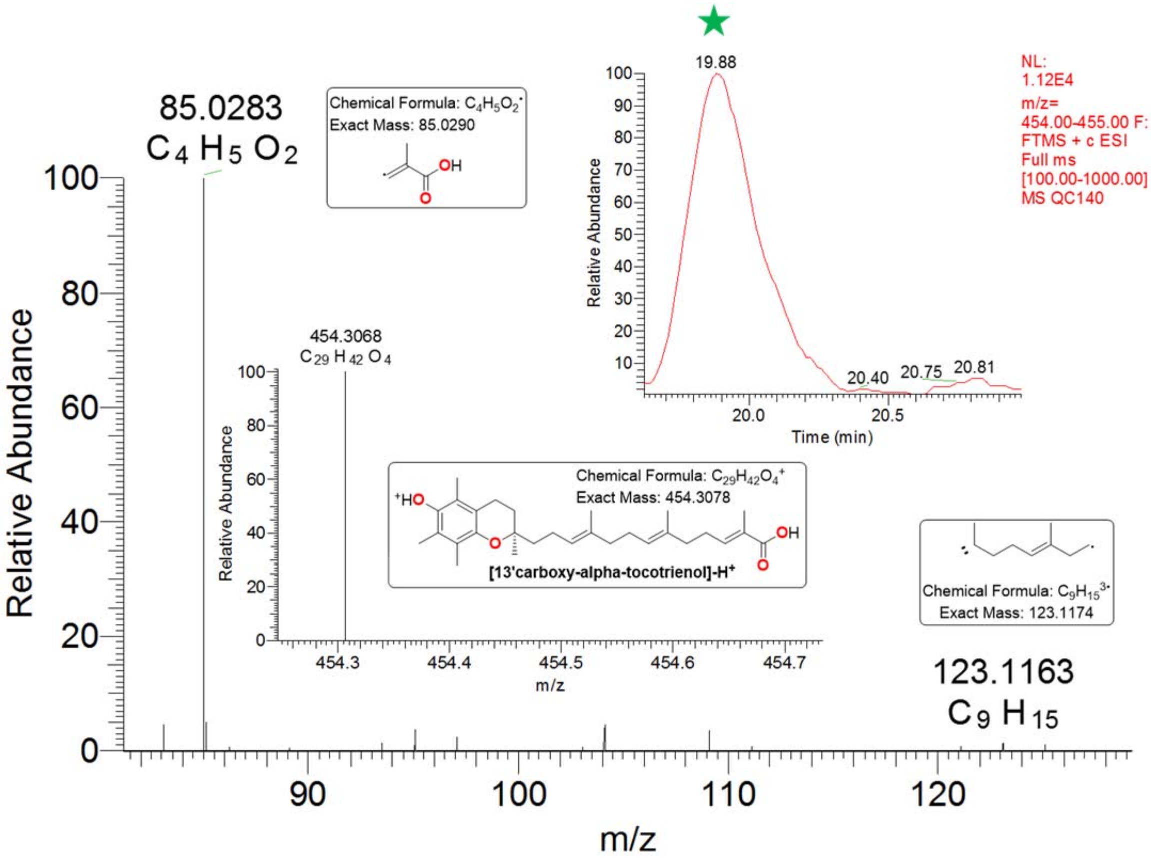


**Supplementary Figure 22|** Accurate mass and MSMS data on 13'-carboxy-alpha-tocotrienol. Raw data used to generate spectra within this image are available within the associated MetaboLights Study upload (MTBLS598).

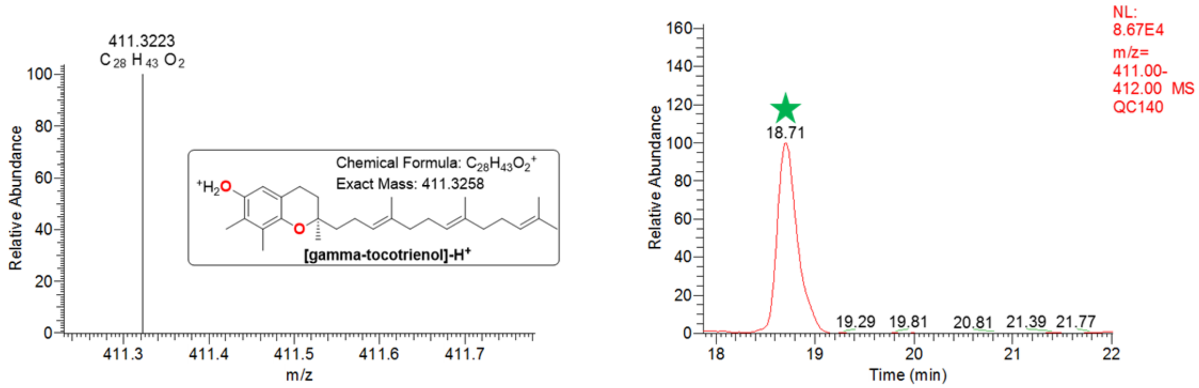


**Supplementary Figure 23|** Accurate mass data on gamma-tocotrienol. Raw data used to generate spectra within this image are available within the associated MetaboLights Study upload (MTBLS598).

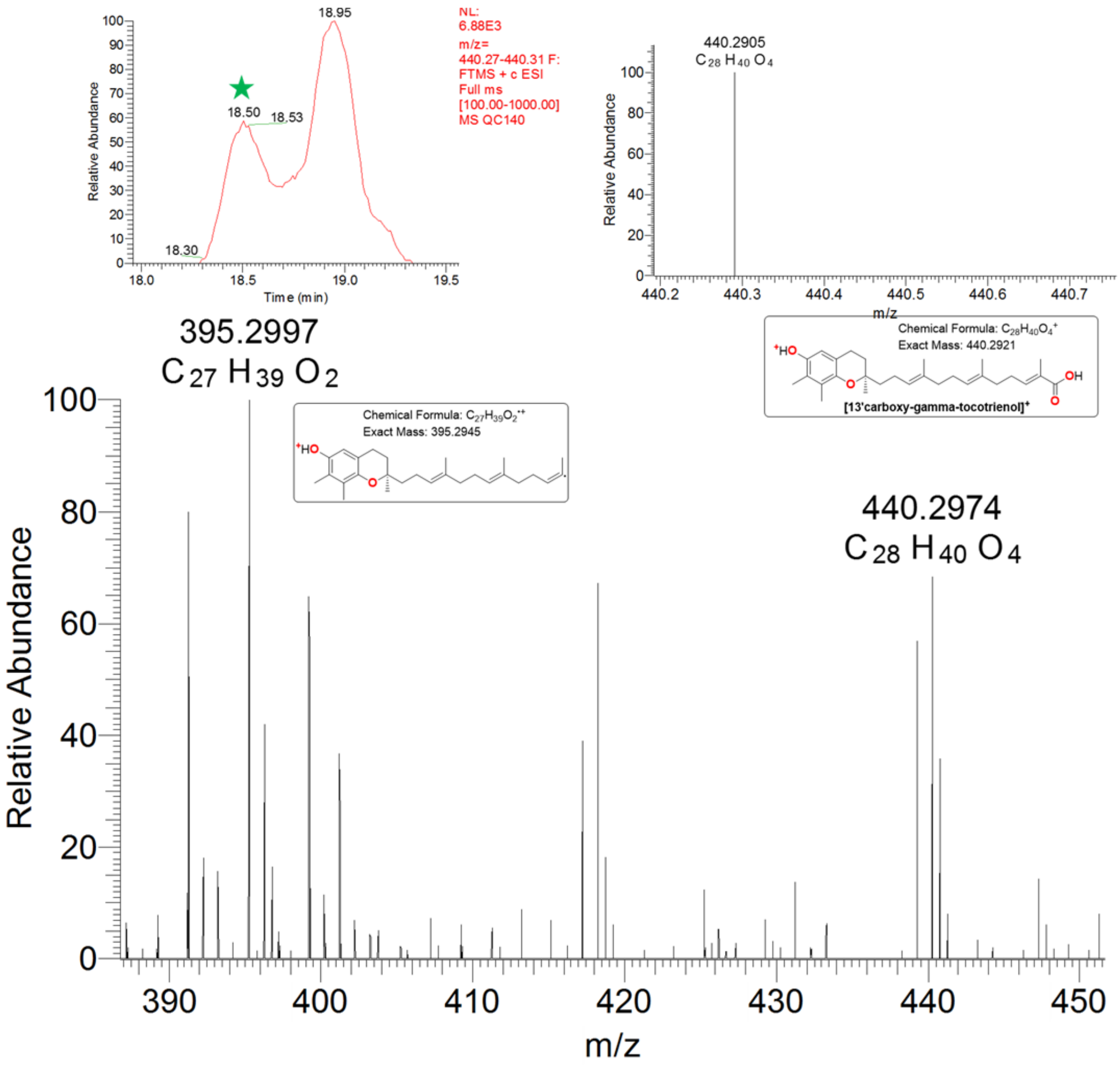


**Supplementary Figure 24|** Accurate mass and MSMS data on 13'-carboxy-gamma-tocotrienol. Raw data used to generate spectra within this image are available within the associated MetaboLights Study upload (MTBLS598).

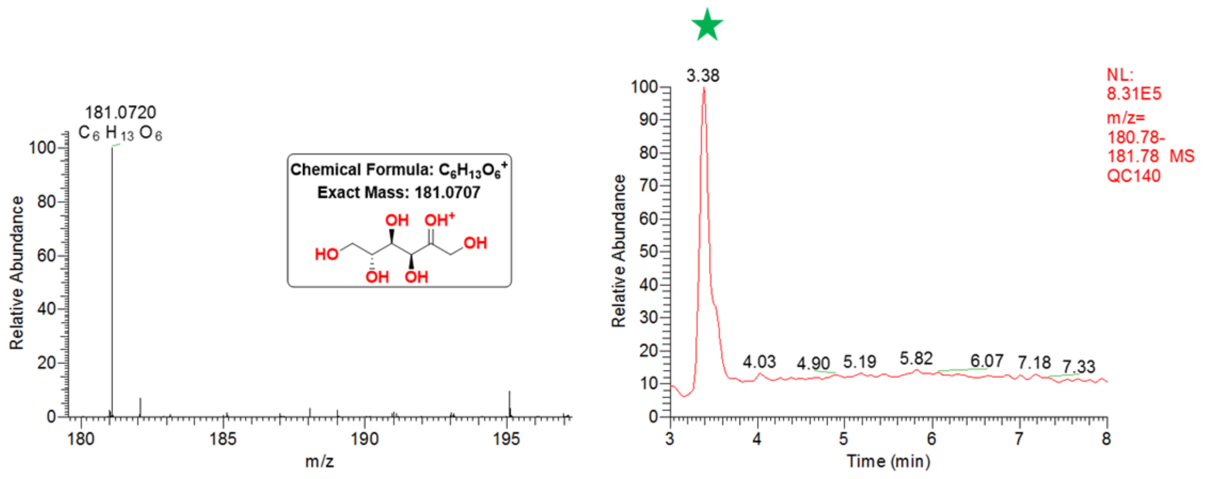


**Supplementary Figure 25|** Accurate mass data on fructose. Raw data used to generate spectra within this image are available within the associated MetaboLights Study upload (MTBLS598).

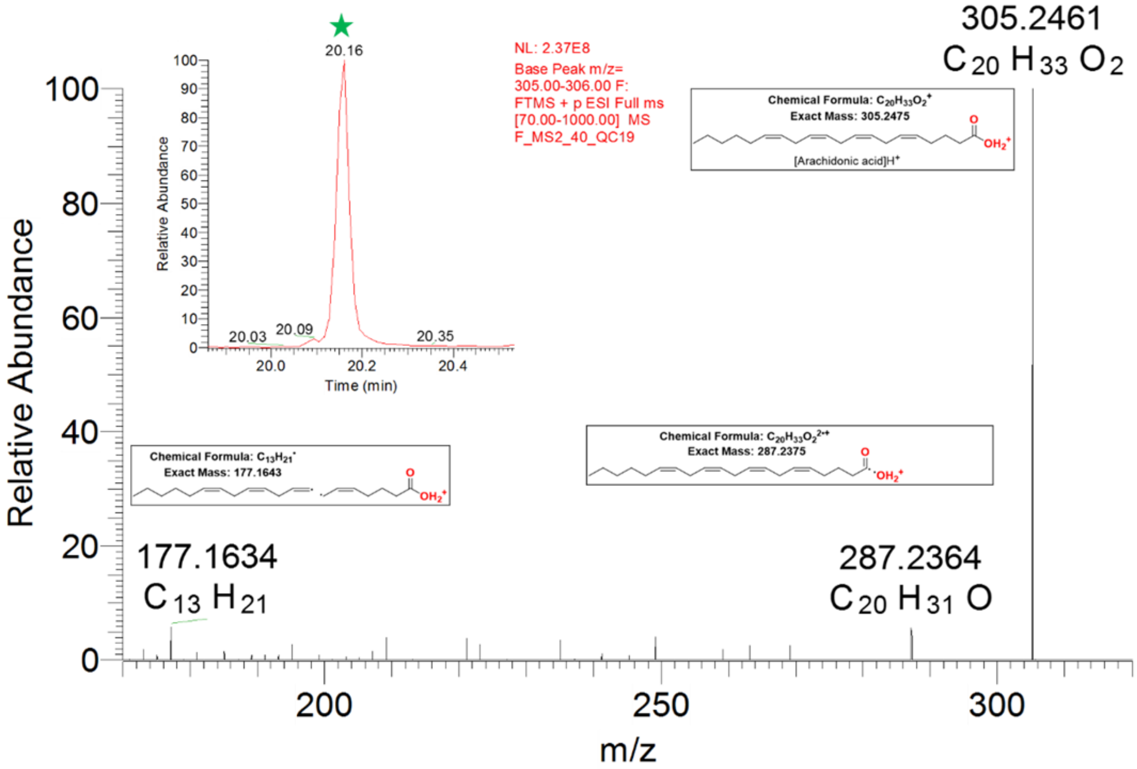


**Supplementary Figure 26|** Accurate mass and MSMS data on arachidonic acid. Raw data used to generate spectra within this image are available within the associated MetaboLights Study upload (MTBLS598).

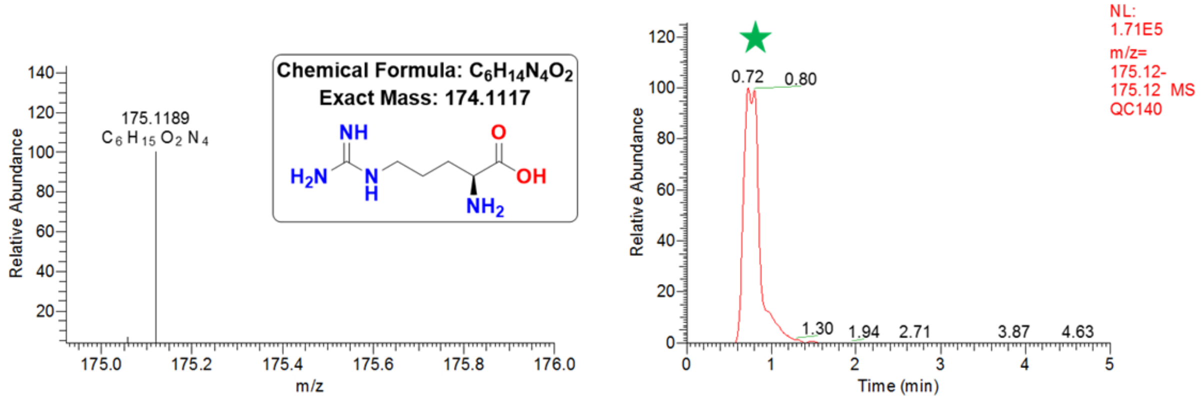


**Supplementary Figure 27|** Accurate mass data on l-arginine. Raw data used to generate spectra within this image are available within the associated MetaboLights Study upload (MTBLS598).

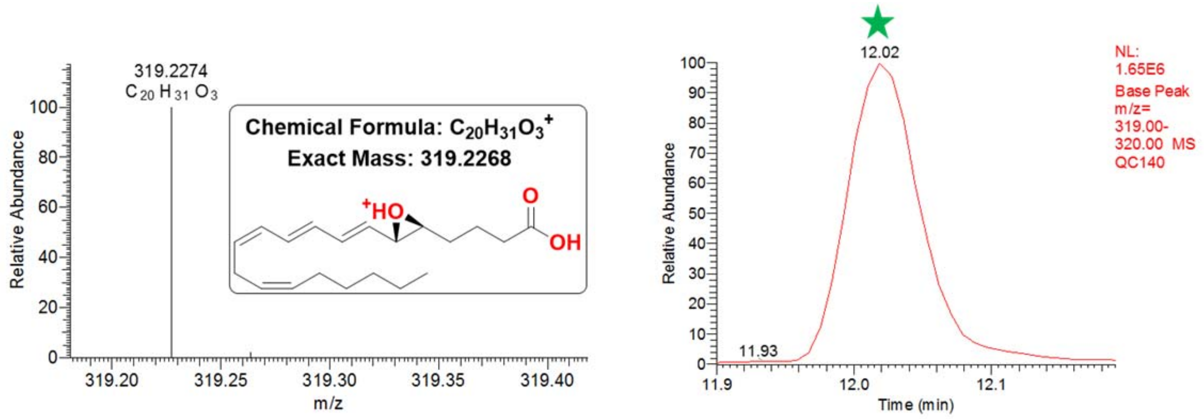


**Supplementary Figure 28|** Accurate mass data on leukotriene A4. Raw data used to generate spectra within this image are available within the associated MetaboLights Study upload (MTBLS598).

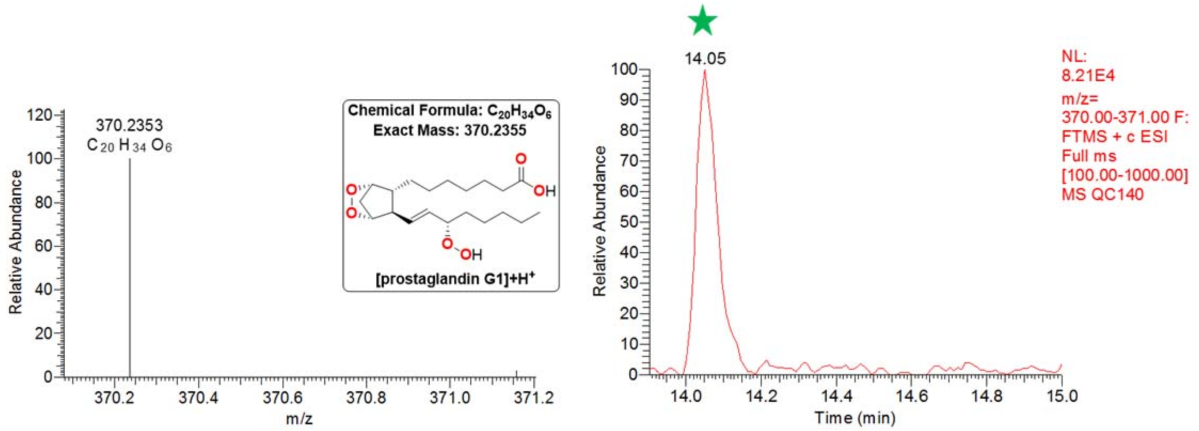


**Supplementary Figure 29|** Accurate mass data on prostaglandin G1. Raw data used to generate spectra within this image are available within the associated MetaboLights Study upload (MTBLS598).

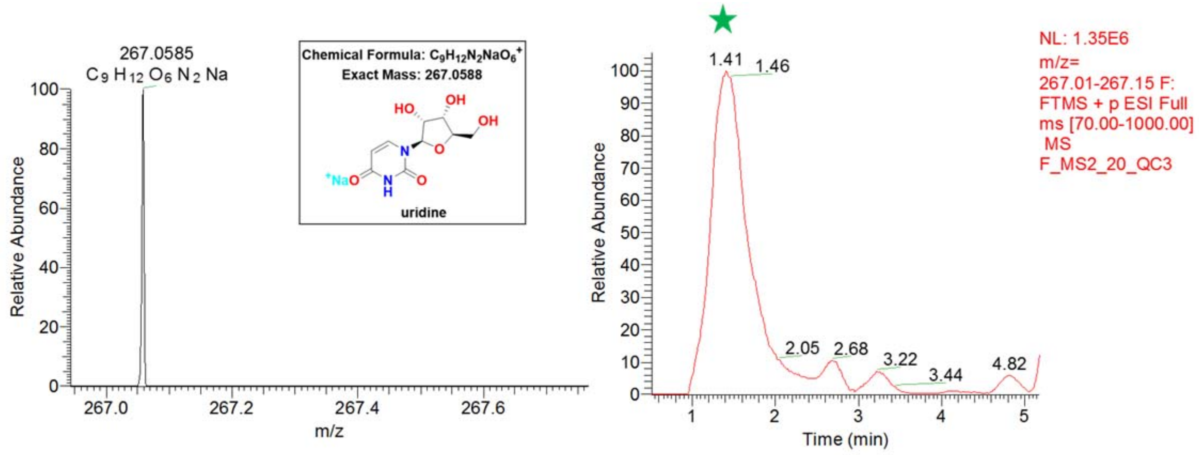


**Supplementary Figure 30|** Accurate mass data on uridine. Raw data used to generate spectra within this image are available within the associated MetaboLights Study upload (MTBLS598).

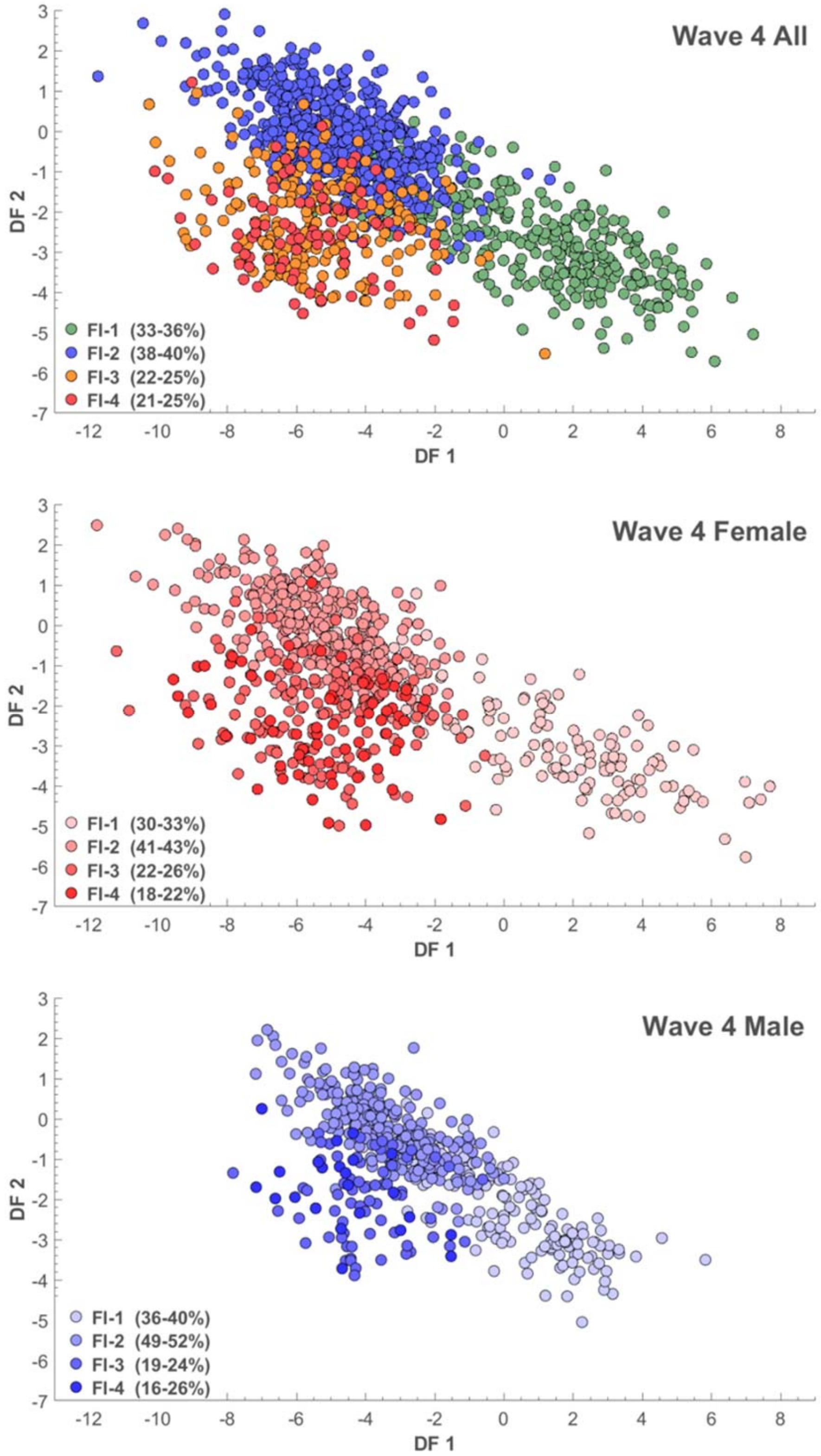


**Supplementary Figure 31|** PC-DFA of Wave 4 UHPLC-MS data stratified by sex. (Green circle = Frailty Index 0-0.1, Blue circle – Frailty Index 0.1-0.2, Orange Circle = Frailty Index 0.2-0.3, Red Circle = Frailty Index above 0.3). Source data are provided as a Source Data file.

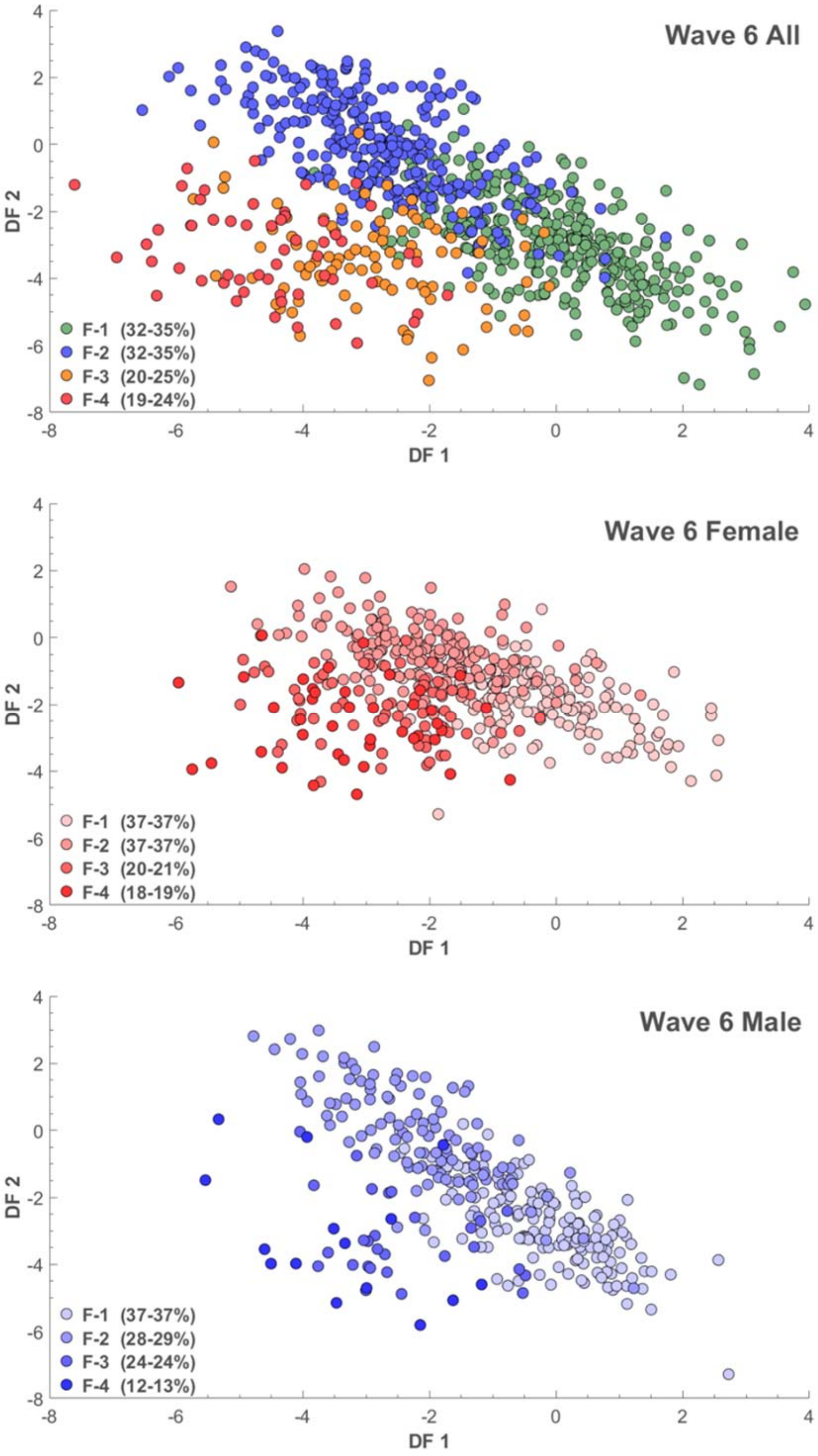


**Supplementary Figure 32|** PC-DFA of Wave 6 UHPLC-MS data stratified by sex. (Green circle = Frailty Index 0-0.1, Blue circle – Frailty Index 0.1-0.2, Orange Circle = Frailty Index 0.2-0.3, Red Circle = Frailty Index above 0.3). Source data are provided as a Source Data file.

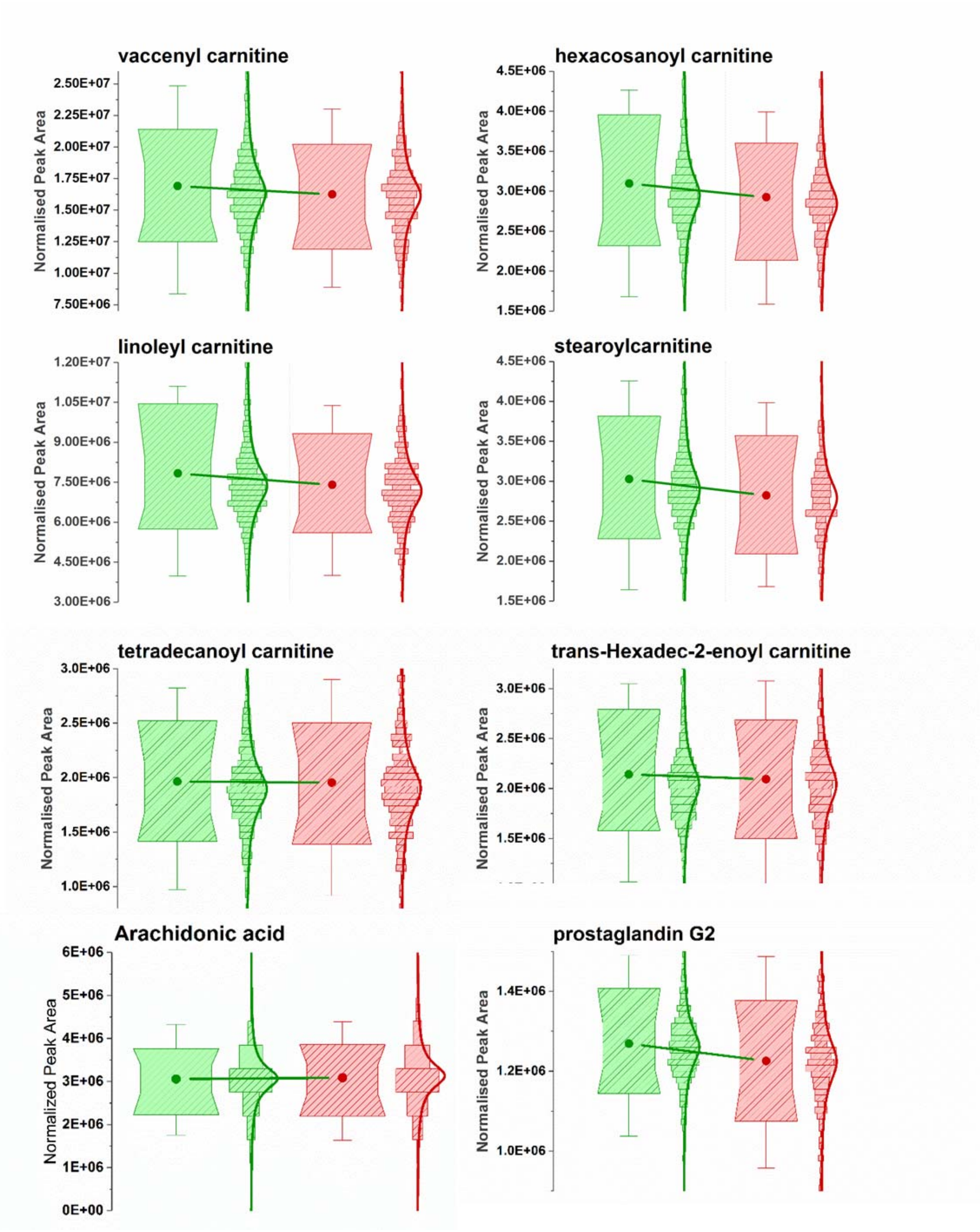


**Supplementary Figure 33|** Metabolite box plots and bin-distributions for carnitine shuttle metabolites indicating abundance directionality from non-frail (green) to frail (red) of metabolites from the carnitine shuttle and vitamin E metabolism. Box limits cover $10^{th}$, $25^{th}$, $75^{th}$ and $90^{th}$ range whilst center dots indicate mean values. Whiskers represent outliers within each dataset. Source data are provided as a Meta Data file.

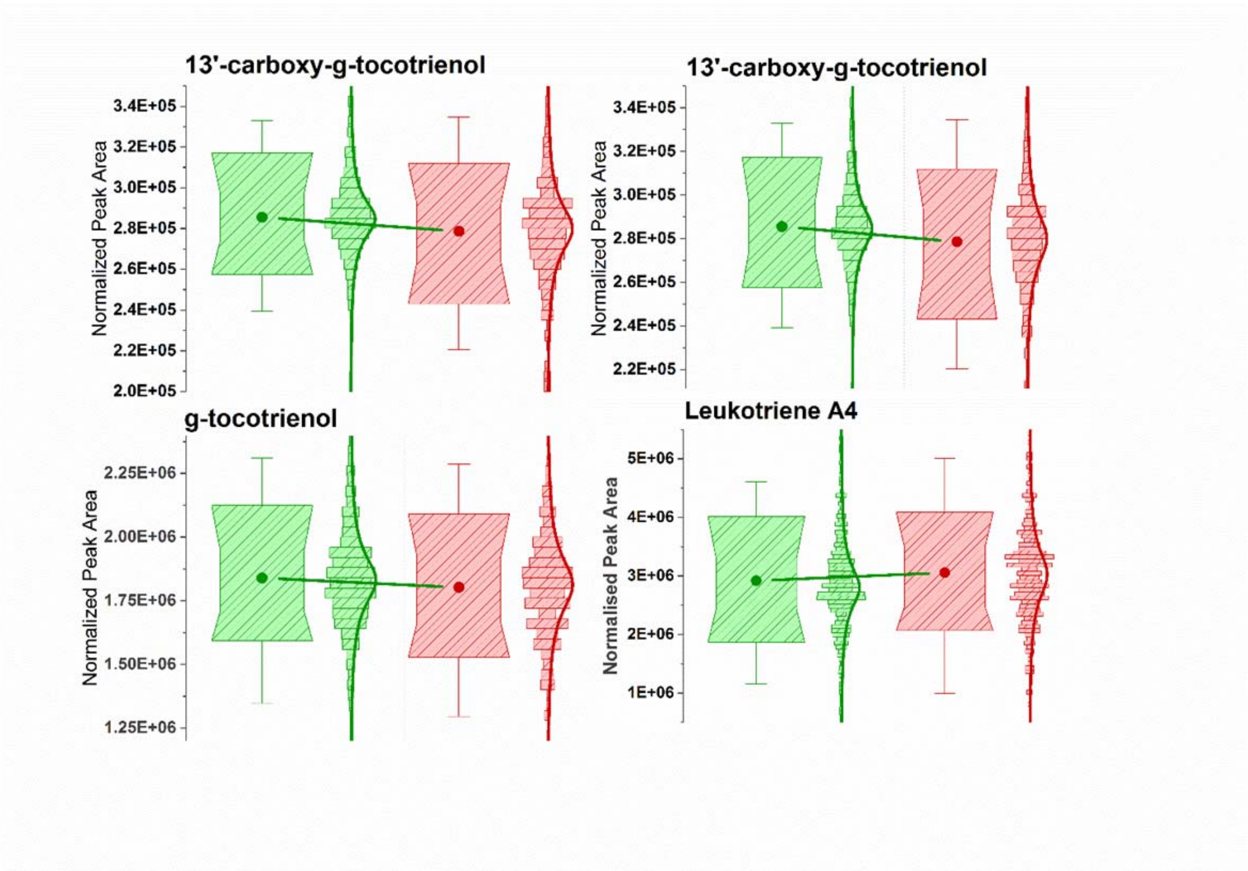


**Supplementary Figure 34|** Metabolite box plots and bin-distributions for vitamin e related metabolites indicating abundance directionality from non-frail (green) to frail (red) of metabolites from the carnitine shuttle and vitamin E metabolism. Box limits cover 10th, 25th, 75th and 90th range whilst center dots indicate mean values. Whiskers represent outliers within each dataset. Source data are provided as a Meta Data file.

**Supplementary Table 1|** 25 Metabolites identified as significant from combined GCMS and LCMS analysis and used in the mummichog pathway analysis to develop the cross-platform metabolic network of frailty.

| Metabolite | Class | Associated Pathway | Analytical Platform | MSI Level | Non-Frail to Frail | Detected in Wave 6 |
|---|---|---|---|---|---|---|
| Glucose | Sugar | Carbohydrate | GCMS | 1 | Up | N/A |
| Citrate | Acid | Carbohydrate | GCMS | 1 | Up | N/A |
| Butyric Acid | Carboxylic Acid | Carbohydrate | GCMS | 1 | Down | N/A |
| Myo-Inositol | Sugar | Carbohydrate | GCMS | 1 | Down | N/A |
| D-Mannose | Sugar | Carbohydrate | GCMS | 1 | Up | N/A |
| Fructose | Sugar | Carbohydrate | GCMS | 1 | Down | N/A |
| Phosphate | Anion | Multiple | GCMS | 1 | Down | N/A |
| L-Arginine | Amino Acid | Multiple | LCMS | 1 | Up | NO |
| Uridine | Nucleoside | Multiple | LCMS | 1 | Down | NO |
| Tryptophan | Tryptophan | Tryptophan | LCMS | 1 | Up | YES |
| 5-Hydroxy-L-tryptophan | Tryptophan | Tryptophan | LCMS | 1 | Up | NO |
| Formyl-5-hydroxykynurenamine | Tryptophan | Tryptophan | LCMS | 2 | Up | YES |
| Formyl-N-acetyl-5-methoxykynurenamine | Tryptophan | Tryptophan | LCMS | 2 | Up | YES |
| Arachidonic acid | Fatty Acid | Carnitine Shuttle | LCMS | 1 | Down | YES |
| Tetradecanoyl carnitine | Carnitine | Carnitine Shuttle | LCMS | 1 | Down | YES |
| trans-Hexadec-2-enoyl carnitine | Carnitine | Carnitine Shuttle | LCMS | 2 | Down | YES |
| Linoleyl carnitine | Carnitine | Carnitine Shuttle | LCMS | 2 | Down | YES |
| Vaccenyl carnitine | Carnitine | Carnitine Shuttle | LCMS | 2 | Down | YES |
| Stearoylcarnitine | Carnitine | Carnitine Shuttle | LCMS | 1 | Down | YES |
| Hexacosanoyl carnitine | Carnitine | Carnitine Shuttle | LCMS | 2 | Down | YES |
| Prostaglandin G2 | Fatty Acid / Hormone | Carnitine Shuttle | LCMS | 2 | Down | NO |
| Leukotriene A4 | Fatty Acid | Vitamin E | LCMS | 2 | Up | NO |
| gamma-tocotrienol | Tocotrienol | Vitamin E | LCMS | 1 | Down | YES |
| 13'-carboxy-gama-tocotrienol | Tocotrienol | Vitamin E | LCMS | 2 | Down | NO |
| 13'-carboxy-alpha-tocotrienol | Tocotrienol | Vitamin E | LCMS | 2 | Down | NO |

**Supplementary Table 2|** Summary of the results of two-sample Mendelian Randomization analysis. The results show a significant association between decreased carnitine levels and frailty (p=0.042, OR=1.53 CI=1.01-2.29) for Exposure 1. SNP=single nucleotide polymorphism, OR=odds ratio, CI=confidence interval.

| Exposure (study) | SNP (gene) | Method | OR of frailty per 10% decrease in carnitine | 95% CI of OR | P value |
|---|---|---|---|---|---|
| 1. Blood metabolite levels (unit increase) (carnitine)[44] | rs12356193 (*SLC16A9*), rs419291 (*SLC22A4*) | Inverse variance weighted | 1.53 | 1.01-2.29 | 0.042 |
| 2.Blood metabolite levels (unit decrease) (carnitine)[44] | rs1466788 (*ALX3*) | Wald ratio | 0.57 | 0.16-2.06 | 0.391 |
| 3.Acylcarnitine levels (unit increase) (Carnitine)[45] | rs1171606 (*SLC16A9*) | Wald ratio | 0.90 | 0.74-1.10 | 0.300 |

**Supplementary References**


1 Tampubolon, G. Trajectories of the healthy ageing phenotype among middle-aged and older Britons, 2004–2013. *Maturitas* **88**, 9-15, (2016).

2 Cheshire H. *et al.* Financial Circumstances, Health and Well-being of the Older Population in England: The 2008 English Longitudinal Study of Ageing Technical Report., (London, 2012).
