# Supplementary material for: Metabolic dysregulation in vitamin E and carnitine shuttle energy mechanisms associate with human frailty
Source: Nat Commun. 2019 Nov 5;10:5027. doi: 10.1038/s41467-019-12716-2 (PMC6831565; doi:10.1038/s41467-019-12716-2)
Supplement: Supplementary file 3 — Reporting Summary [file 41467_2019_12716_MOESM3_ESM.pdf]

nature research

Corresponding author(s): Dr Nicholas JW Rattray

Last updated by author(s): 2019/08/23

Reporting Summary

Nature Research wishes to improve the reproducibility of the work that we publish. This form provides structure for consistency and transparency in reporting. For further information on Nature Research policies, see Authors & Referees and the Editorial Policy Checklist.

Statistics

For all statistical analyses, confirm that the following items are present in the figure legend, table legend, main text, or Methods section.

n/a	Confirmed	
☐	☒	The exact sample size (*n*) for each experimental group/condition, given as a discrete number and unit of measurement
☐	☒	A statement on whether measurements were taken from distinct samples or whether the same sample was measured repeatedly
☐	☒	The statistical test(s) used AND whether they are one- or two-sided<br>*Only common tests should be described solely by name; describe more complex techniques in the Methods section.*
☐	☒	A description of all covariates tested
☐	☒	A description of any assumptions or corrections, such as tests of normality and adjustment for multiple comparisons
☐	☒	A full description of the statistical parameters including central tendency (e.g. means) or other basic estimates (e.g. regression coefficient) AND variation (e.g. standard deviation) or associated estimates of uncertainty (e.g. confidence intervals)
☐	☒	For null hypothesis testing, the test statistic (e.g. *F*, *t*, *r*) with confidence intervals, effect sizes, degrees of freedom and *P* value noted<br>*Give P values as exact values whenever suitable.*
☒	☐	For Bayesian analysis, information on the choice of priors and Markov chain Monte Carlo settings
☒	☐	For hierarchical and complex designs, identification of the appropriate level for tests and full reporting of outcomes
☐	☒	Estimates of effect sizes (e.g. Cohen's *d*, Pearson's *r*), indicating how they were calculated

Our web collection on statistics for biologists contains articles on many of the points above.

Software and code

Policy information about availability of computer code

Data collection	The main source of data collected within this study was mass spectrometry raw data. For LCMS data generation and instrument operation Xcaliber Software version 3.0 (Thermo-Fisher Scientific, Germany) was used. For GCMS data generation and instrument operation chromaTOF Software (Leco Inc. USA).
Data analysis	PC-DFA, PLS-DA, PLS-R and robust spline alignment analysis were carried out using MATLAB 2012a (MathWorks, Natick, MA, USA) using our in-house bioinformatics toolbox cluster-toolbox-v2.0 and can be accessed through the Github repository (https://github.com/biospec).<br>Prior to chemometric analysis data matrices were log-2-transformed to account for skewed distribution in Excel 2015<br>Linear regressions models and diagnostic plots were performed using R-Studio (Version 1.0.44) using the lm function. Univariate t-tests, cross validation and heat-map correlation were performed using MetaboAnalyst 3.0 (http://www.metaboanalyst.ca/).

For manuscripts utilizing custom algorithms or software that are central to the research but not yet described in published literature, software must be made available to editors/reviewers. We strongly encourage code deposition in a community repository (e.g. GitHub). See the Nature Research guidelines for submitting code & software for further information.

Data

Policy information about availability of data

All manuscripts must include a data availability statement. This statement should provide the following information, where applicable:

- Accession codes, unique identifiers, or web links for publicly available datasets
- A list of figures that have associated raw data
- A description of any restrictions on data availability

All metadata, mass spectrum files and statistical packages used in this paper are freely available and deposited in accessible public repositories. All English Longitudinal Study of Ageing (ELSA) data files are available from the United Kingdom Data Service repository
http://discover.ukdataservice.ac.uk/catalogue?sn=5050
Mass Spectrum and metabolomics data are accessible through the EMBL-EBI MetaboLights repository - www.ebi.ac.uk/metabolights/ Study Identifier MTBLS598

Statistical scripts used to perform PC-DFA, PLS-R and PLS-DA were developed within the www.biospec.net cluster-toolbox and are freely available on the open source GitHub repository hosted at github.com/Biospec/cluster-toolbox-v2.0

The source data underlying Figs 1,2,3,4 & 5 and Supplementary Figs 1-15 and 31-34 are provided within the supplied Source Data file alongside data used to generate the Z-scores. Supplementary Figs 16-30 were generated in the Thermo Fisher Xcaliber Software using the raw LCMS data available within the upload supplied to the MetaboLights repository.

All data are available from the corresponding author upon reasonable request.

Field-specific reporting

Please select the one below that is the best fit for your research. If you are not sure, read the appropriate sections before making your selection.

☒ Life sciences ☐ Behavioural & social sciences ☐ Ecological, evolutionary & environmental sciences

For a reference copy of the document with all sections, see nature.com/documents/nr-reporting-summary-flat.pdf

Life sciences study design

All studies must disclose on these points even when the disclosure is negative.

Sample size	The determination of a priori sample size estimation in metabolomics is difficult due to the highly collinear nature of multivariate data – that is groups of metabolites (classes or those on the same pathway) can project in a similar direction making it very hard to pull apart and statistically determine the individually significant features that separate groups, without high sample number to pull signals from the baseline noise. For untargeted serum work looking to generate hypotheses on population data, once you start reaching 400-600 samples your accuracy begins to level out – if you have approx. 500 serum samples per class then you should begin to get in a statistical area that where you are in and about a confidence interval of 95%. This concept is documented within the literature in the attached Dunn – Metabolomics 2015 paper (refer to fig 3). Within our experimental design we proposed two groups (frail and non-frail) and thus 1200 samples is an appropriate figure. As this work has been designed as a population level assessment of frailty, an important strength of the study is its unselected nature which greatly increases the external validity of the findings.
Data exclusions	Not applicable.
Replication	Biologically, one of the strengths of our approach has been to reproduce our analyses on a subset of 760 serum samples taken from the same subjects, 4 years later. We assessed metadata from 1753 subjects in this longitudinal manner and carried out identical metabolomics analysis on a subset of these.
Randomization	Subjects were not initially designated in to experimental groups, but placed on an indexed scale via a clinical scoring mechanism. Frail/pre-frail/non-frail groups were subsequently designated by results from multivariate analysis alongside comparison to index scores from the literature. Analytically, all samples were randomized before being allocated in to run order blocks.
Blinding	Not applicable as groupings were not identified until post-analysis.

Reporting for specific materials, systems and methods

We require information from authors about some types of materials, experimental systems and methods used in many studies. Here, indicate whether each material, system or method listed is relevant to your study. If you are not sure if a list item applies to your research, read the appropriate section before selecting a response.

Materials & experimental systems

n/a	Involved in the study	
☒	☐	Antibodies
☒	☐	Eukaryotic cell lines
☒	☐	Palaeontology
☒	☐	Animals and other organisms
☐	☒	Human research participants
☒	☐	Clinical data

Methods

n/a	Involved in the study	
☒	☐	ChIP-seq
☒	☐	Flow cytometry
☒	☐	MRI-based neuroimaging

Human research participants

Policy information about studies involving human research participants

Population characteristics	The Fig 1 and MetaData tabs of the supplied Source Data File contains 47 different descriptors that document main characteristics of the 1191 participants (including age/height/weight/gender/blood analyses).

Recruitment	Participants were recruited via advertisement with local doctor's surgeries/hospitals within the north west of England. This fact ofrestricted geography of the cohort requires that further validation from a range of independent cohorts is essential to test the conserved nature of the results. This is highlighted within the discussion section of the article.
Ethics oversight	The London Multi-Centre Research Ethics Committee approved the sample/study collection protocol. This is highlighted within the manuscript.

Note that full information on the approval of the study protocol must also be provided in the manuscript.
